# *Pelargonium sidoides* radix extract EPs 7630 reduces rhinovirus infection through modulation of viral binding proteins on human bronchial epithelial cells

**DOI:** 10.1371/journal.pone.0210702

**Published:** 2019-02-01

**Authors:** Michael Roth, Lei Fang, Daiana Stolz, Michael Tamm

**Affiliations:** Pulmonary Cell Research & Pneumology, Department Biomedicine & Department of Internal Medicine, University & University Hospital Basel, Basel, Switzerland; Centre National de la Recherche Scientifique, FRANCE

## Abstract

Bronchial epithelial cells are the first target cell for rhinovirus infection. The course of viral infections in patients with acute bronchitis, asthma and COPD can be improved by oral application of *Pelargonium sidoides* radix extract; however, the mechanism is not well understood. This study investigated the *in vitro* effect of *Pelargonium sidoides* radix extract (EPs 7630) on the expression of virus binding cell membrane and host defence supporting proteins on primary human bronchial epithelial cells (hBEC). Cells were isolated from patients with severe asthma (n = 6), moderate COPD (n = 6) and non-diseased controls (n = 6). Protein expression was determined by Western-blot and immunofluorescence. Rhinovirus infection was determined by immunofluorescence as well as by polymerase chain reaction. Cell survival was determined by manual cell count after live/death immunofluorescence staining. All parameters were determined over a period of 3 days. The results show that EPs 7630 concentration-dependently and significantly increased hBEC survival after rhinovirus infection. This effect was paralleled by decreased expression of the inducible co-stimulator (ICOS), its ligand ICOSL and cell surface calreticulin (C1qR). In contrast, EPs 7630 up-regulated the expression of the host defence supporting proteins β-defensin-1 and SOCS-1, both in rhinovirus infected and un-infected hBEC. The expression of other virus interacting cell membrane proteins such as MyD88, TRL2/4 or ICAM-1 was not altered by EPs 7630. The results indicate that EPs 7630 may reduce rhinovirus infection of human primary BEC by down-regulating cell membrane docking proteins and up-regulating host defence proteins.

## Introduction

Bronchial epithelial cells (BEC) are the main target of rhinovirus infection, which is the most frequent cause of common cold as well as exacerbation in patients with asthma and COPD [1–3]. Exacerbations are the main cause of disease severity and progression [1,2]. Rhinovirus infection correlates with the seasonal frequency of exacerbations in asthma and COPD patients and it was suggested that preventive measures reducing viral infection would benefit these patients [4, 5].

EPs 7630, a proprietary aqueous-ethanolic extract from *Pelargonium sidoides* roots, has been shown to shorten viral infections. It is widely used in the treatment of acute airway infections and has been investigated as an add-on therapy for asthma and COPD patients. However, the mechanism of the protective effect of EPs 7630 is not completely understood. EPs 7630 significantly reduced bacterial and viral infection by immunomodulatory actions [6, 7]. In childhood asthma, a 5 day application of EPs 7630 significantly improved symptoms of a viral infection of the upper respiratory tract [8, 9]. In acute bronchitis, a 7-day course with EPs 7630 improved symptoms and significantly increased the proportion of patients able to attend kindergarten, school or work on day 7 compared to placebo [10, 11]. EPs 7630 has demonstrated antiviral effects against a broad panel of respiratory viruses [12].

Besides directly eliminating viruses, it may be possible to prevent infection by reducing the expression of proteins that enable the virus to dock to the cell surface [5, 13]. In regards to lung diseases, such preventive anti-viral strategies have not been investigated. However, in other conditions rhinovirus infection was reduced by blocking ICAM-1, suggesting that this molecule functions as a docking protein and supports infection [14, 15]. In addition, ICAM-1 mediated rhinovirus-induced inflammation in asthma and COPD [16, 17]. However, the study by *Mukhopadhyay et al* [16] was not supporting the hypothesis that ICAM-1 is necessary for rhinovirus infection as described for COPD [14, 17].

The inducible costimulatory (ICOS) and its ligand ICOSL are expressed by human BEC (hBEC) and therefore may support the function of antigen presenting immune cells [18]. In other lung epithelial cell types ICOS expression was linked to ICAM-1 and major histocompatibility protein-1 mediating antigen presentation [19, 20]. Concerning asthma, ICOSL stimulated airway smooth muscle cell proliferation and thus may contribute to hyperplasia in asthma [21]. In the epithelial cell membrane several proteins, summarized as collectins, are expressed and modify the immune response to infections [22]. These proteins have to form complexes, which signal through C1qR (cell surface calreticulin, ecto-calreticulin, calregulin). Interestingly, cell surface C1qR has been reported to bind enteroviruses and to induce phagocytosis, thereby increasing anti-viral cell response in other cell types than epithelial cells [23, 24]. In human bronchial epithelial cells bacterial cell extracts up-regulated C1qR expression by activation of extracellular mitogen activated protein kinases 1/2 [25]. In human immune cells, EPs 7630, reduced the expression of virus stimulated secretion of TNF-α and IL-10, while it up-regulated IL-6 (interferon β2) through p38 mitogen activated protein kinases [26]. However, a link of EPs 7630 treatment and virus binding proteins has not been investigated for rhinovirus in the context of asthma and COPD.

Rhinovirus infection was reduced when the host cell expressed β-defensin [27]. Immuno-modulating bacterial lysates up-regulated the expression of β-defensin-2 in the mucosa of human volunteers, but the consequence for viral infections was not determined [28]. However, the observation that β-defensin-1 was highly expressed by COPD patients, puts the potential protective role of β-defensins into question [29], as COPD patients frequently suffer from exacerbations due to viral infection including rhinovirus [2]. In childhood asthma the use of bacterial extracts increased the expression of β-defensin which correlated with reduced exacerbation/infection rates [30].

Rhinovirus infection was increased when suppressor of cytokine signalling 1 (SOCS1) was up-regulated by transforming growth factor β in BEC, suggesting an anti-viral effect of SOCS1 [31]. In asthma, SOCS1 expression negatively correlated with RV16 or RV1 induced cytokine secretion by BEC [32]. Thus, the results of the latter study also indicate that different strains of rhinovirus may have distinct effects on this host defence mechanism. In monocytes, rhinovirus up-regulated SOCS1 expression on the mRNA level, which correlated with reduced secretion of anti-inflammatory cytokines such as CXCL10 and IFN-α [33]. Therefore, the role of SOCS1 in response to rhinovirus infection may be cell type specific and should be further investigated.

In this study, we assessed the effect of EPs 7630 on the expression of cell membrane proteins, which may play a role in rhinovirus infection, and on anti-viral host defence proteins in primary hBEC of patients with asthma or COPD as well as in non-diseased controls.

## Material and methods

### Patients

The study cohort consisted of healthy probands (n = 6), asthma (n = 6), and COPD (n = 6) patients. Healthy controls have been selected from patients undergoing diagnostic bronchial biopsy for other reasons than asthma or COPD. The ethical approval to obtain the required tissue samples was approved by the local ethical committee, EK Nordwest und Zentralschweiz (EKNZ BASEC 2016–01057) and each patient gave written informed consent.

Tissues were obtained from either non-diseased lung tissue obtained from patients undergoing lung resection for lung metastasis cancer therapy (Thoracic Surgery, University Hospital Basel, Basel, Switzerland).

Asthma patients were classified as Mild–sever according to GINA guidelines (Pneumology Clinics, University Hospital Basel, Basel, Switzerland).

COPD patients were classified according to GOLD guidelines (Pneumology Clinics, University Hospital Basel, Basel, Switzerland)

Patients with acute exacerbation or known viral/bacterial infection were excluded.

Tissue donor characteristics are displayed in Table 1.

**Table 1:** Patient details (FEV1: forced exhaled volume over 1 second; FCV: forced vital capacity; NA: not available).

diagnosis	age	gender	FEV1 (%)	FCV (%)	mediaction (2 weeks before biopsy)
**controls**					
**1**	**45**	**male**	**NA**	**NA**	**NA**
**2**	**75**	**male**	**134**	**128**	**none**
**3**	**61**	**female**	**NA**	**NA**	**none**
**4**	**75**	**male**	**98**	**106**	**none**
**5**	**32**	**female**	**94**	**97**	**none**
**6**	**39**	**male**	**71**	**74**	**none**
**Mean ± SEM**	**54.5 ± 8.3**		**99.2 ± 11.6**	**101.2 ± 9.9**	
**asthma**					
**1**	**51**	**male**	**106**	**124**	**ICS + LABA**
**2**	**57**	**female**	**29**	**75**	**ICS + LABA**
**3**	**20**	**feamle**	**58**	**73**	**ICS + LABA**
**4**	**66**	**female**	**34**	**79**	**ICS + LABA**
**5**	**19**	**female**	**69**	**70**	**ICS + LABA**
**6**	**67**	**male**	**61**	**85**	**ICS + LABA**
**Mean ± SEM**	**46.7 ± 9.8**		**59.5 ± 12.4**	**84.3 ± 8.9**	
**COPD1**					
**1**	**76**	**male**	**20**	**46**	**ICS + LABA**
**2**	**54**	**male**	**49**	**76**	**ICS + LABA**
**3**	**74**	**male**	**36**	**69**	**ICS + LABA**
**4**	**73**	**female**	**57**	**82**	**ICS + LABA**
**5**	**60**	**female**	**76**	**128**	**ICS + LABA**
**6**	**53**	**male**	**42**	**90**	**ICS + LABA**
**Mean ± SEM**	**65.0 ± 4.7**		**46.7 ± 8.5**	**81.8 ± 12.1**	

### Drug

**EPs 7630** was supplied by Schwabe Pharma AG, 6403 Küssnacht am Rigi. The compound was dissolved in cell culture medium to the final concentrations before being applied to the cell cultures.

### BEC isolation and characterisation

This has been published earlier [25, 34]. Small pieces of bronchial tissues (1 x 1 x 1 mm) were placed into cell culture vessels, which were pre-wetted with BEC specific medium Cnt-PR-A (CellnTech, Bern, Switzerland). The medium was replaced every second day and cells were passaged by mechanical shaking of dividing cells. Cells were characterised by positive staining of E-Cadherin and Pan-Keratin, and negative staining for fibronectin [25].

### Ratio live/death cells

Cells were treated with either EPs 7630 and/or infected with RV for 24 hours and were then stained a live for the ratio of live (blue)/dead (green) cells according to the manufacturer’s instructions (Molecular Probes, Mammalian cell viability, Thermofisher Scientific, Switzerland). Cells were incubated for 30 minutes with the double stain solution at 37oC and then monitored and photographed by EVOS cell imaging system (Thermofisher Scientific). The ratio of green to blue cells was counted and calculated as percentage of total cells.

### Rhinovirus (RV) infection and determination of infection rate

The RV strain used for the experiments has been described earlier and was identified as RV16 [35]. For the experiments described below, we have used the same stock described earlier [35].

**Fig 1:**
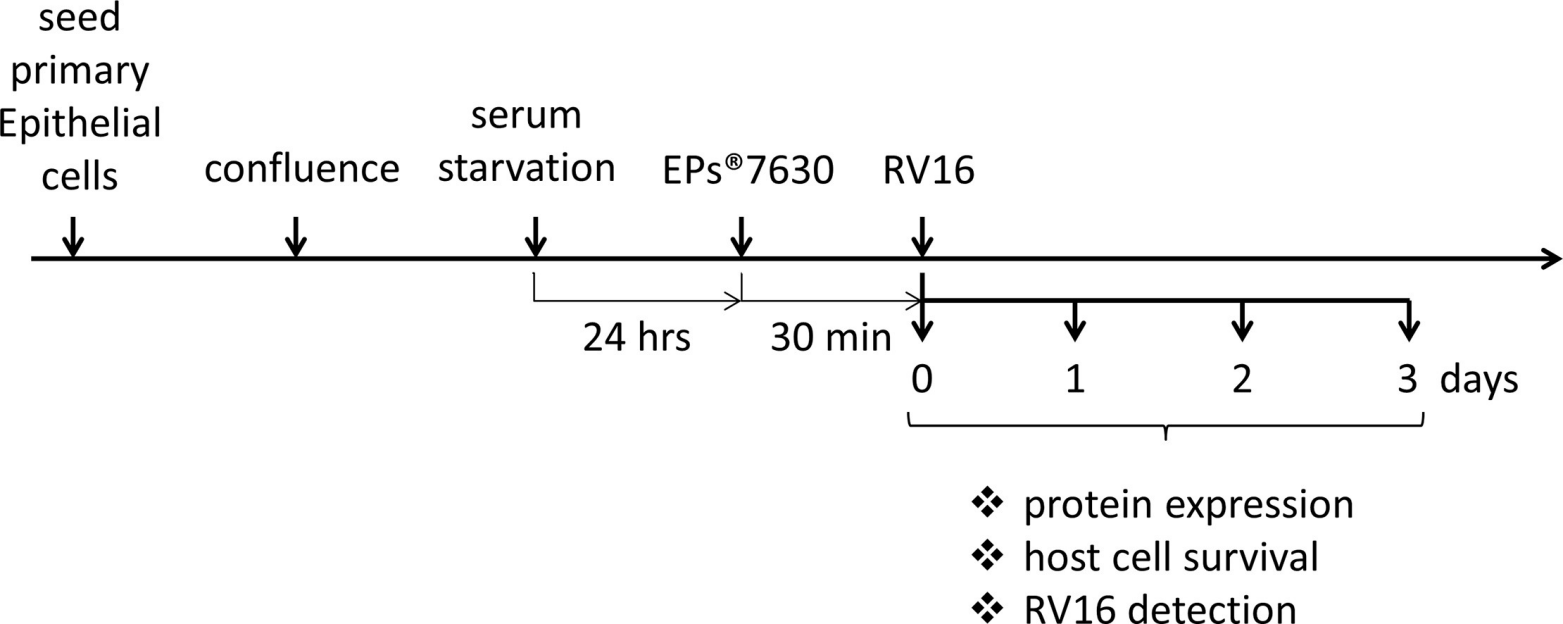
Treatment of hBEC with EP 7630 and RV16 infection.

HBEC were infected with 1x multiplicity of infection (MOI) of RV16 for up to 3 days. The infection rate was determined by immunofluorescence staining using an anti-RV16 antibody (cat# 18758, QED-Bioscience Inc. San Diego, USA). Cells were seeded into 8-well chamber slides (Thermofisher Scientific, Switzerland), and treated with either RV16 and/or EPs 7630, as indicated in the treatment schemata provided in Fig 1.

After treatment, the cells were fixed in 4% formalin (in PBS) for 5 minutes after washing with PBS. The fixed cells were washed 2x with PBS and permeabilised for 15 minutes with 0.01% TWEEN-100 in PBS. Unspecific binding was blocked with 2% bovine serum albumin (30 minutes in PBS) before being incubated overnight (4 ^o^C) with the anti-RV16 antibody (1:100 dilution). Following 3 washes with PBS, cells were incubated with an anti-mouse FITC labelled antibody (Abcam, Switzerland) for 1 hour at room temperature. After 3 washes with PBS, the number of RV16 positive cells was counted by immunofluorescence microscopy (EVOS FLoid cell imaging station, Thermofisher Scientific) and nuclei were stained for cell counting using the distributor’s live cell reagent (Thermofisher Scientific).

### RV- polymerase chain reaction

Polymerase chain reaction was performed with the following primers: RVqR447f: 5’-GGC CCC TGA ATG YGG CTA A-3’ qR561r: 5’-GAA ACA CGG ACA CCC AAA GTA G-3’, following the protocol described by others [36]. The PCR products were analysed by gel electrophoresis.

### Immuno-blotting

Immuno-blotting for protein analysis has been described earlier [25]. Following protein separation through a gradient polyacrylamide gel (4–12%) and electro-blotting onto a PVDF membrane, proteins were identified and their expression rate determined by Western-blotting. The following proteins were detected: β-defensin-1, C1qR, ICAM1, ICOS, ICOSL, MYD88, and SOCS (all Abcam, Cambridge, UK). Details of dilution and producers are provided in Table 2.

**Table 2:** Antibodies used for protein analysis. Abcam, Cambridge, U.K.

Antigen	Immuno-blot dilution	Imuno-fluorescence	ELISA &	Producer	Cat-#
C1qR	1:5,000	1:100	1:2000	Abcam	ab134079
ICAM1	1:1,000	1:100	1:2000	Abcam	ab2213
β-defensin	1:1,000	1:100	1:2000	Abcam	ab14425
MYD88	1:5,000	1:50	1:1000	Abcam	ab2068
ICOS	1:5,000	1:50	1:1000	Abcam	ab133680
ICOSL	1:5,000	1:50	1:1000	Abcam	ab138354
SOCS	1:5‘000	1:100	1:1000	Abcam	Ab9870

Membranes were blocked for 1 hour (room temperature) in PBS containing 0.01% Tween-20 and 2% bovine serum albumin. The primary antibodies were added at concentrations indicated in Table 1 and incubated overnight at 4°C. Following 3 washes with blocking buffer, membranes were incubated with secondary species specific antibodies labelled with horse radish peroxidase for 1 hour. Unbound antibody was washed off by 3 washes with blocking buffer and protein bands were visualised by exposure to X-ray films.

GAPDH expression was used to normalize protein expression and estimated changes induced by the different treatments.

### Semi-quantitative cell based ELISA for cell surface proteins

Confluent cells were treated either with EPs 7630 or RV-16 or a combination for 24 hrs. Cells were then washed once with PBS and fixed for 2 x 5 minutes in 4% paraformaldehyde. 30 minutes after blocking the cells in PBS-tween20 (0.1%) and 2% bovine serum albumin the cell were incubated overnight (4°C) with one of the above listed antibodies Table 2. Cells were then washed 3 x with PBS and incubated with a horseradish labelled secondary species specific antibody for 2 hours at room temperature. After additional washes with PBS bound antibodies were detected by adding 100 μl TMB-substrate for 15 minutes and the reaction was topped by adding 50 μl of 0.1 N HCl. The absorbance was read at 490 nm and the data is displayed as optical density arbitrary units. The method had been described earlier [37].

### Immunofluorescence (IF)

For immunofluorescence analysis, cells were seeded in 8-well chamber slides (cat. 94.6140.802 Sarstedt AG, Sevelen, Switzerland). Following 24 hours serum deprivation cells were pre-treated with EPs 7630 for 60 minutes, followed by infection with RV16 (1 MOI/ml) for various time points (0, 1, 2, or 3 days). At the end of the infection period cells were fixed in 4% paraformaldehyde in PBS for 2 x 5 min. and immuno-fluorescence staining was performed as described previously [14]. Primary antibodies (Table 1) were diluted 50-100-fold in 2% BSA-PBS and incubated at 4°C overnight, followed by 3 washes and subsequent staining with a PE-labelled goat anti-rabbit IgG secondary antibody or FITC-labelled goat anti-mouse IgG secondary antibody (Santa Cruz Biotechnology Inc, CA). Nuclei were stained by DAPI. Images were captured by EVOS FLoid cell imaging station and nuclei were stained for cell counting using the distributor’s live cell reagent (NucBlue^TM^, cat R37605, Thermofisher Scientific).

### Statistics

The Null-hypothesis was: No effect of EPs 7630 on the expression of cell surface protein expression, rhinovirus infection or cell survival. Treated cells were compared to non-treated cells or rhinovirus infected cells. P-values were calculated by ANOVA or Student’s t-test (paired, two-tailed) and p-values < 0.05 were considered as significantly different from controls.

## Results

Using human primary bronchial epithelial cells and smooth muscle cells (control: n = 6; asthma: n = 6, COPD: n = 6) the effect of EPs 7630 on the expression of virus docking proteins and rhinovirus infection was assessed. The scheme for treatment of primary epithelial cells is shown in Fig 1.

### EPs 7630 reduces RV16 infection and improves survival of host cells

**Fig 2:**
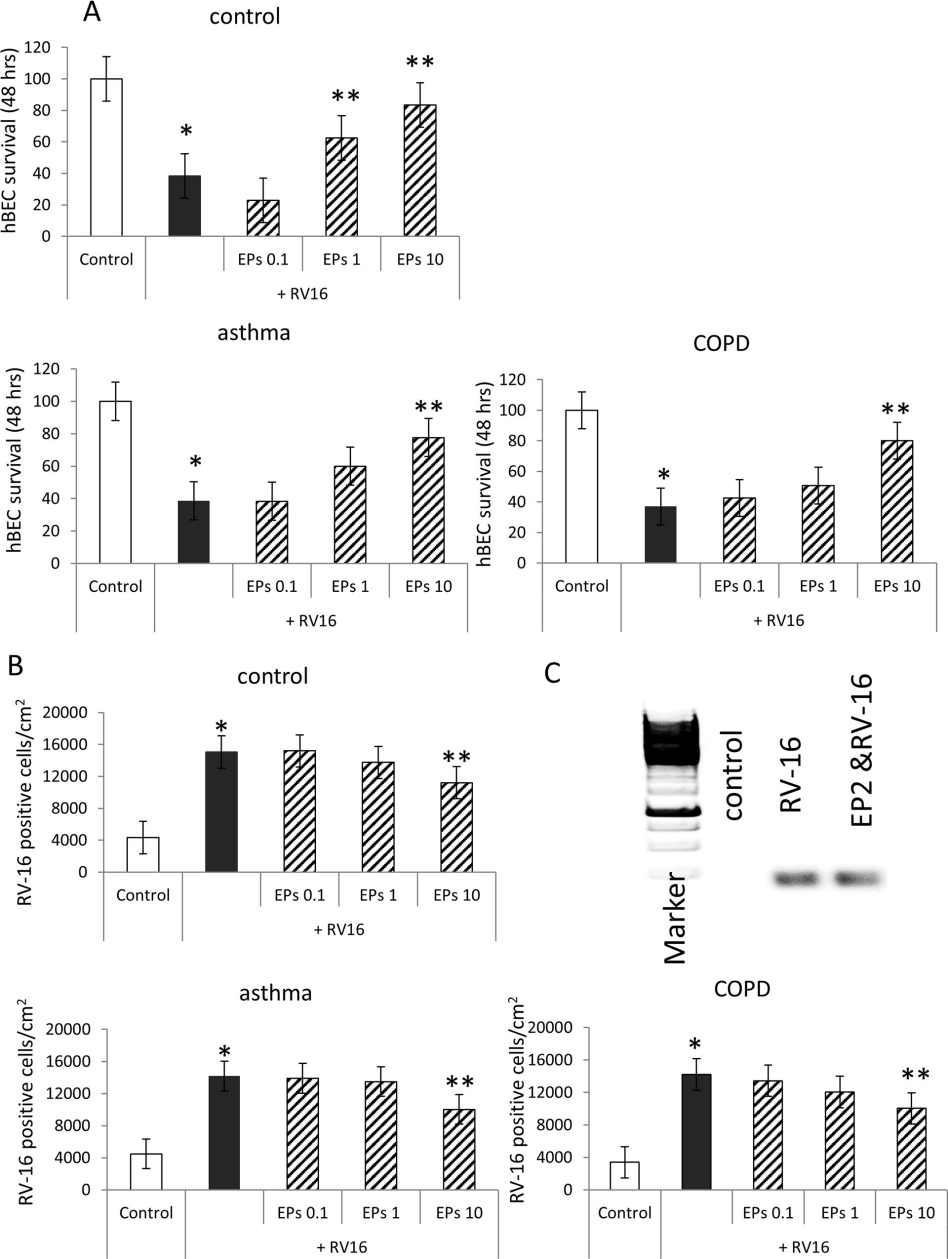
(A) Concentration-dependent preventive effect of EPs 7630 on hBEC number at 2 days after RV16 infection. (B) Concentration dependent reduction of RV16 positive (green) hBEC after 2 days. Bars represent mean ± S.E.M. of six cell lines in each group. Statistics were calculated as Student’s paired t-test. * indicates P<0.05 compared to untreated hBEC (control) and **indicate p<0.05 compared to RV16 infected cells. Insert: PCR product for RV-16 in epithelial cells in the presence and absence of EPs 7630 over 24 hours. Complete data of survival and RV-positive cells is provided in the supporting information (Figs A and B in S1 File, Tables A and B in S2 File).

Epithelial cells were incubated with one infectious unit (IU) of RV16 for 24, 48, and 72 hours, after which the infection was determined by immunofluorescence staining for RV16. Infections periods shorter than 48 hours did not show significant effects on survival and RV16 positive hBEC. RV16 infection significantly reduced survival of hBEC over 48 hours and this effect was reduced when hBEC had been pre-incubated with EPs 7630 in a concentration dependent manner (Fig 2A). No disease specific effect was observed on the rescue effect of EPs 7630 or on RV16 infection.

RV16 infection at 48 hours is depicted as representative phase-contrast microscopy photographs in Fig 2A. Before infection or drug treatment epithelial cells showed characteristic knobble-stone phenotype (Fig A in S1 File, t = 0). EPs 7630 alone (Fig A in S1 File, second row) did not alter the phenotype over 72 hours, while RV16 infection significantly reduced cell numbers (Fig A in S1 File, 3^rd^ row, 1^st^ photograph). When pre-incubated for 24 hours with EPs 7630, cell survival was increased after RV16 infection. RV16 infection was concentration-dependently reduced by EPs 7630 (Fig A in S1 File, 3^rd^ & 4^th^ row).

RV16 infection was also monitored by immune-fluorescence staining for RV16 and the intensity of the staining was quantified by a computer assisted image analysis program (Fig 2B). Furthermore, we depict a representative PCR-gel for the reduced expression of RV- RNA after 24 hours treatment with Eps 7630 (Fig 2B, insert). The results of RV16 -immunostaining showed a significant reduction of RV16 following 48 hours pre-incubation with EPs 7630 (Fig 2B). This protective effect was EPs 7630 was increasing with time and became significant for lower concentrations at 72 and 96 hrs (Fig B in S1 File). No disease specific effect was observed comparing the effect of EPs 7630 between control, asthma and COPD cells.

In Fig 2C, we show a representative PCR-product gel for the reduce RV-16 expression after 24 hours treatment with EPs 7630.

### EPs 7630 reduced the expression of viral docking proteins on host cells

**Fig 3:**
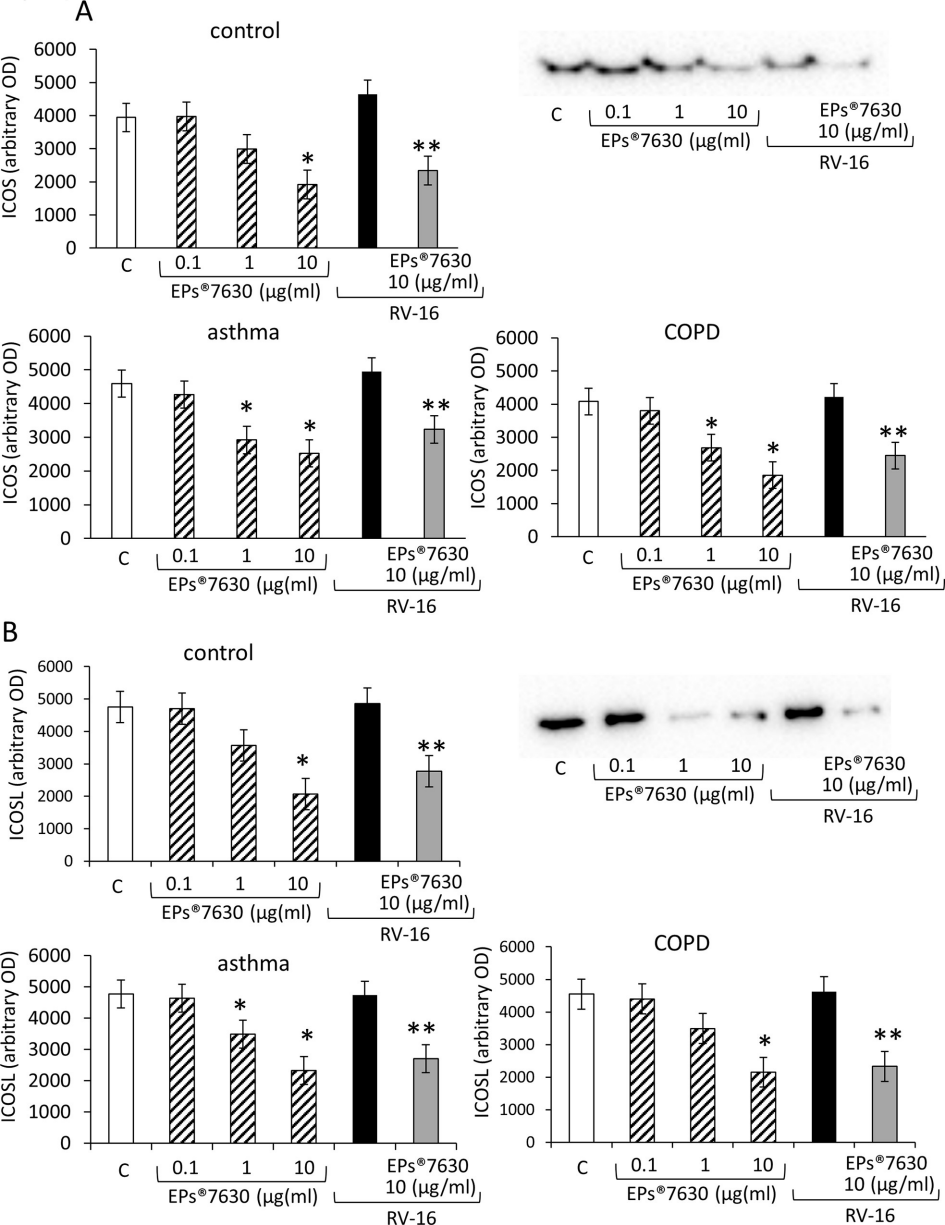
Effect of EPs 7630 on ICOS (A) and ICOSL (B) expression by human primary bronchial epithelial cells following 1 day incubation. Bars represent OD. mean ± S.E.M.of six independent experiments in different cells lines of each group. “C” indicates untreated hBEC; * indicate P-value <0.05 compared to control; ** indicates P-value < 0.05 compared to RV16 infected hBEC. “C” indicates untreated hBEC; * indicate P-value <0.05 compared to control; ** indicates P-value < 0.05 compared to RV16 infected hBEC. Representative Immuno-blots are presented as inserts to Fig 3A and 3B and complete data of immuno-blots are shown in the supporting information (Tables A and B and Fig C in S3 File).

In primary hBEC pre-treatment with EPs 7630 for 24 hours had distinct effect on the expression of viral docking proteins. As shown in Fig 3A, EPs 7630 decreased the expression of ICOS below the base line level of untreated hBEC as determined by Western-blotting and subsequent image analysis of protein bands. The effect of the compound was concentration dependent and also occurred in hBEC which were infected by RV16 24 hours after treatment with EPs 7630 (Fig 3A). There was no significant difference of the overall beneficial effect of EPs 7630 in the three groups of hBEC. However, controls needed a 10x higher concentration of EPs 7630 to reduce ICOS expression (Fig 3A). RV16 infection alone slightly increased the expression of ICOS compared to untreated cells, but this effect was not significant (Fig 3A).

Similar to ICOS, the expression of its ligand ICOSL was reduced by EPs 7630 in a concentration dependent manner in all three hBEC groups (Fig 3B). The reducing effect of EPs 7630 on ICOSL expression was stronger in hBEC of asthma patients. RV16 infection had no effect on the expression of ICOSL (Fig 3B). The reducing effect of EPs 7630 (10 μg/ml) on ICOSL expression was also observed in RV16 infected hBEC (Fig 3B). Representative Immuno-blots are presented as inserts to Fig 3A and 3B and complete Immuno-blots are shown in the supporting information (Tables A and B and Fig C in S3 File).

**Fig 4:**
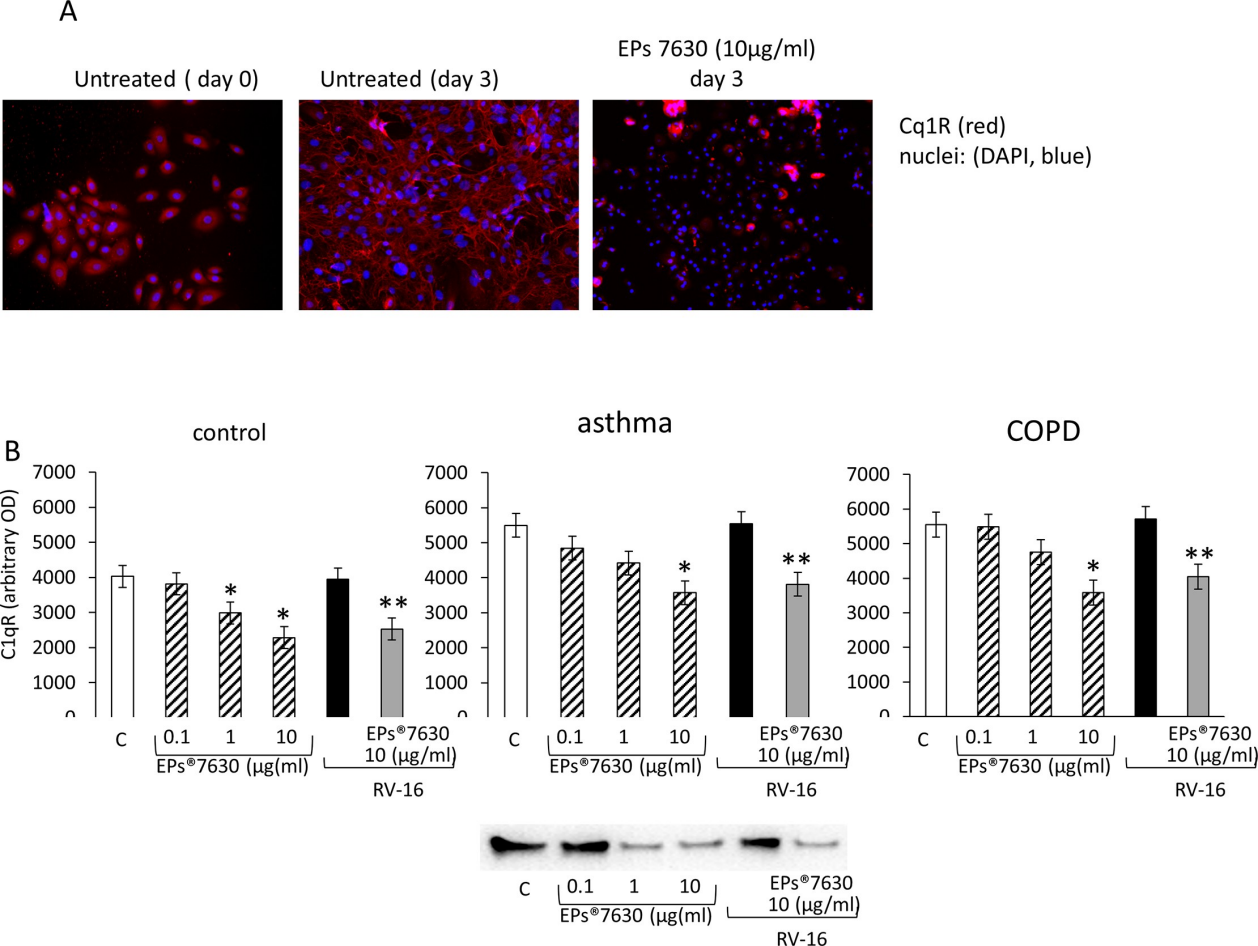
(A) Representative immune-cytochemistry of C1qR localisation in the absence and presence of EPs 7630 or RV16 infection in primary human hBEC of healthy controls. Similar results were obtained in hBEC obtained from patients with moderate-severe asthma or COPD. (B) Semi-quantitative image analysis (ImageJ) of C1qR in six Western-blots in each donor group. Bars represent OD. mean ± S.E.M.of six independent experiments in different cells lines of each group. “C” indicates untreated hBEC; * indicate P-value <0.05 compared to control; ** indicates P-value < 0.05 compared to RV16 infected hBEC. Representative Immuno-blots are presented as inserts to Fig 4A and 4B and complete data of immuno-blots are shown in the supporting information (Table A and Fig B in S4 File).

Depicted by fluorescence microscopy, untreated hBEC expressed C1qR in the cytosol and the cell membrane (Fig 4A, t0). Three days after treatment with EPs 7630, the expression of C1qR was reduced and the remaining protein shifted into the cytosol (Fig 4A, second panel). RV16 infection stimulated the accumulation of C1qR in the cellular membrane in the majority of hBEC (Fig 4A, third panel). The reducing effect of EPs 7630 on C1qR and the relocation was not affected by RV16 infection (Fig 4A, fourth panel). The reducing effect of EPs 7630 on C1qR expression was confirmed by Western-blotting which showed a concentration dependent effect (Fig 4B). Western-blot image analysis suggested an increased C1qR expression in hBEC of asthma and COPD donors compared to healthy tissue donors. However, the overall inhibition of C1qR by EPs 7630 was documented in all groups (Fig 4B). Representative Immuno-blots are presented as inserts to Fig 4A and 4B and complete Immuno-blots are shown in the supporting information (Table A and Fig B in S4 File).

### EPs 7630 increased the expression of anti-viral host defence proteins

**Fig 5:**
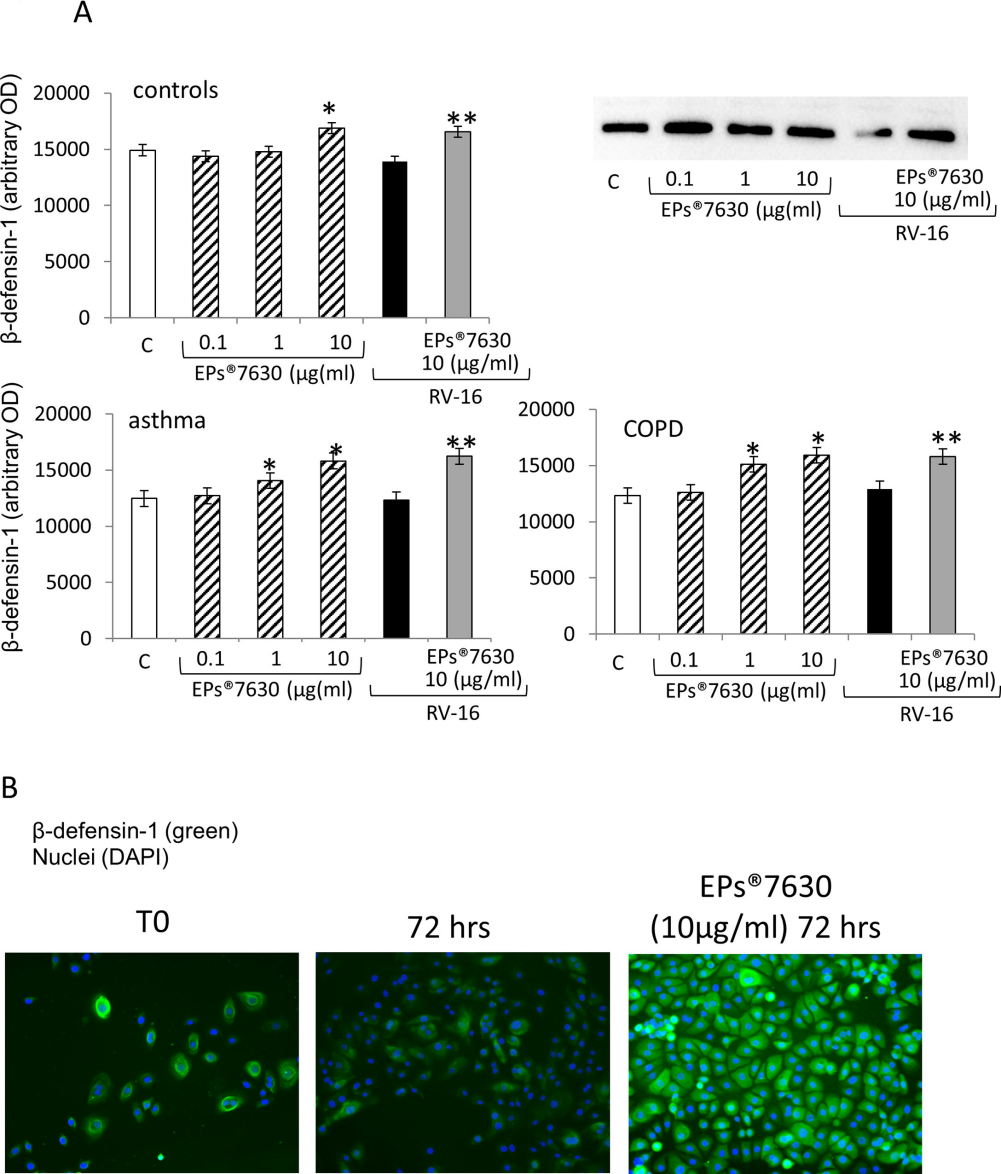
(A) One day incubation with EPs 7630 stimulated the expression of the anti-viral cell surface protein β-defensin-1. “C” indicates untreated hBEC; * indicate P-value <0.05 compared to control; ** indicates P-value < 0.05 compared to RV16 infected hBEC. (B) Representative immunofluorescence photographs of β-defensin-1 expression in primary human epithelial cells in the presence and absence of EPs 7630 at three days in primary human hBEC of healthy controls. Similar results were obtained in hBEC obtained from patients with moderate-severe asthma or COPD. Bars represent OD. mean ± S.E.M.of six independent experiments in different cells lines of each group. “C” indicates untreated hBEC; * indicate P-value <0.05 compared to control; ** indicates P-value < 0.05 compared to RV16 infected hBEC. Representative Immuno-blots are presented as inserts to Fig 5A and 5B and complete data of immuno-blots are shown in the supporting information (Table A and Fig B in S5 File).

RV16 has been reported to affect the expression of β-defensin-2 and -3 as described above, but no data is available of its effect on β-defensin-1. In hBEC, RV16 infection did not significantly affect the expression of β-defensin-1 over a period of three days, while EPs 7630 concentration dependent increased its expression (Fig 5A). When hBEC were pre-treated with EPs 7630 and afterwards infected with RV16, the expression of β-defensin-1 was still up-regulated in all three donor groups (Fig 5A). The stimulatory effect of EPs 7630 on β-defensin-1 was stronger in cells obtained from patients with asthma or COPD, compared to controls (Fig 5A). The increase of β-defensin-1 by EPs 7630 could also be detected by immunofluorescence microscopy as shown in Fig 5B.

**Fig 6:**
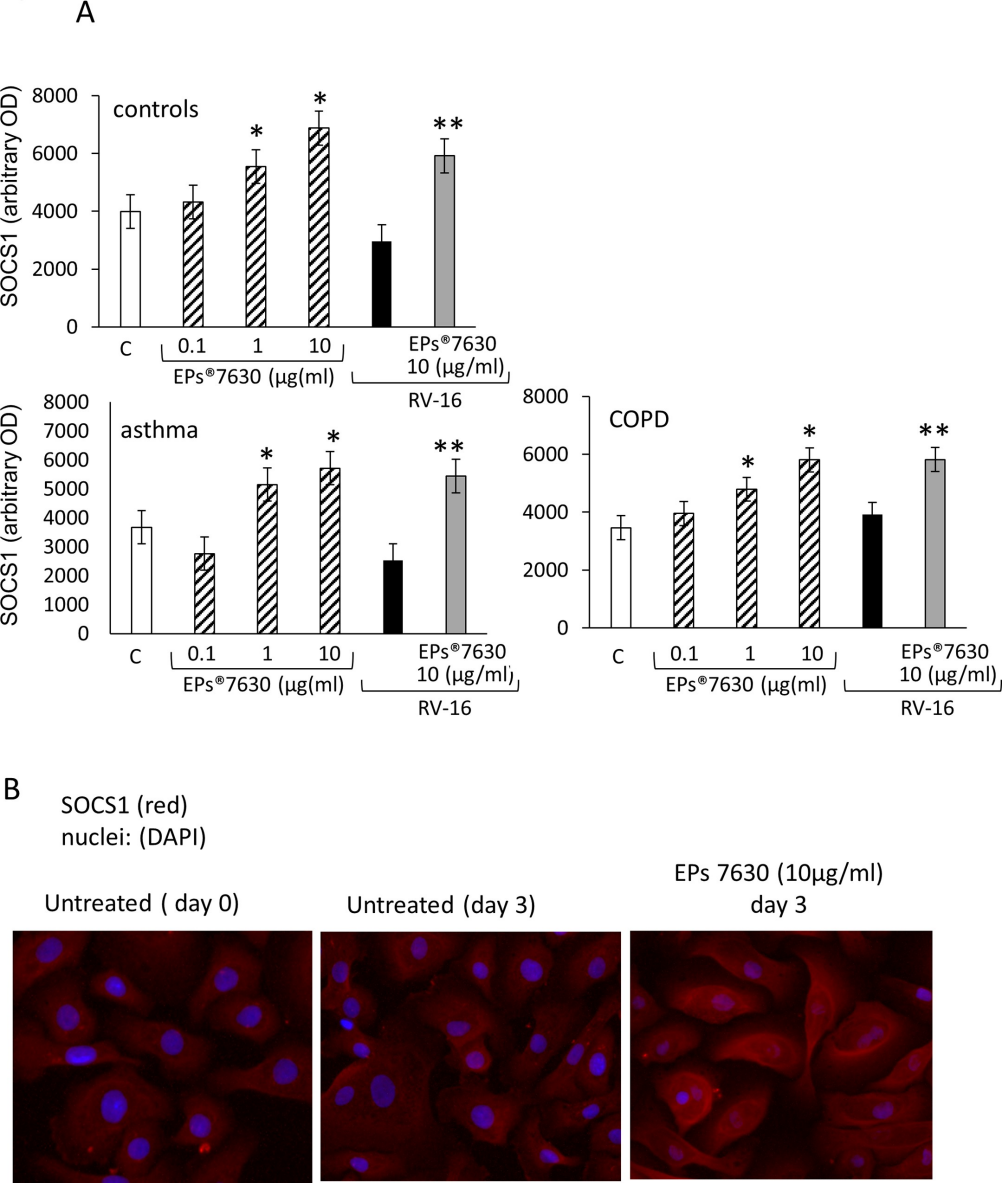
(A) Concentration dependent induction of SOCS-1 by EPs 7630 in the presence and absence of RV16 infection. Bars represent mean ± S.E.M. of six cell lines in each group. “C” indicates untreated hBEC; * indicate P-value <0.05 compared to control; ** indicates P-value < 0.05 compared to RV16 infected hBEC. Data of immuno-blot analysis are presented in supporting information (S6 File). (B) Representative immunofluorescence image of SOCS1 at 48 hours in the presence and absence of EPs 7630 in primary human hBEC of healthy controls. Similar results were obtained in hBEC obtained from patients with moderate-severe asthma or COPD.

EPs 7630 increased the expression of a second host defence protein, SOCS1 in a concentration dependent manner as shown in Fig 6A. The increase of SOCS1 by EPs 7630 was similar in all three hBEC groups (Fig 6A). RV16 infection slightly reduced the expression of SOCS1 but the effect was not significant compared to untreated hBEC (Fig 6A). When cells were pre-treated for 24 hours with EPs 7630, the infection with RV16 had no significant effect on the increased expression of SOCS1 as shown in Fig 6A. Fluorescence microscopy confirmed the increase of SOCS1 in hBEC exposed to EPs 7630 for more than 24hours (Fig 6B).

## Discussion

The presented data suggest that EPs 7630 reduces rhinovirus infection by modification of hBEC function. The beneficial effect of EPs 7630 on rhinovirus infection may be in part explained by the net-effect of the compound on reducing potential rhinovirus docking proteins and simultaneously increasing the expression of host defence systems. Thus, the infection rate will be reduced and intracellular killing of rhinovirus may be increased.

Pre-incubation with EPs 7630 significantly reduced the signal for RV16 infection in epithelial cells indicating that it reduces virus replication. These findings are in line with reports showing that EPs 7630 reduced the replication of influenza-A virus, respiratory syncytial virus, corona virus, parainfluenza virus and coxsackie virus [12]. The reduction of viral replication by EPs 7630 may explain the beneficial effects of the drug on acute respiratory tract infection in children [38], and the reduced frequency of exacerbation in COPD patients [39].

Cell membrane C1qR has been reported to mediate virus phagocytosis of enteroviruses which led to intracellular killing [23,24]. Up-regulation of C1qR after 3 days treatment with a bacterial extract was reported earlier in human bronchial epithelial cells and was linked to reduced RV-16 infection [25]. In this study we observed that EPs 7630 down-regulated C1qR over three days in the same cell type. These results may be opposing, but both may lead to reduced virus infection. As long as the role of C1qR in viral infection is unclear, increased intracellular killing after phagocytosis as well as reduced docking of RV might result in lower infection of the host cells.

ICOS and its ligand ICOSL have been discussed as possible targets or tools to improve virus defence [38]. The fact that ICOS and ICOSL are expressed by different types of lung epithelial cells has been reported by others [18, 20, 40]. However, this is the first report demonstrating that expression of ICOS and ICOSL is reduced by an immune modulator such as EPs 7630 also when the cells were infected with RV16. This effect of EPs 7630 occurred together with the reduced infection rate of RV16, however, we have no proof in this study that both events are linked to each other.

In alveolar cells and hBEC, respiratory syncytial virus up-regulated ICOS expression and it was assumed that ICOS functions as a binding protein for viruses [18]. For other enteroviruses the increased expression of ICOS was reported on immune cells [41]. In other organs, epithelial cell specific expression of ICOS and ICOSL affected immune cell function and thereby contributed to host response [42]. The inhibition of ICOS and ICOSL have been discussed for some time also in regard to asthma associated host immune tolerance, however, yet a therapeutic application has to be developed [43].

The blockade of ICOS and ICOSL signalling by synthetic inhibitors such as ICOSLg have been reported in other diseases and in animal models [44, 45]. In both reports, the inhibition of ICOS/ICOSL significantly reduced inflammation. Furthermore, ICOSL stimulated proliferation of airway smooth muscle cells and its inhibition may reduce airway wall remodelling in asthma [21]. Thus, the down-regulated expression of ICOS and ICOSL on hBEC by EPs 7630 may have two beneficial effects in chronic inflammatory lung diseases: (i) it reduces the ability of rhinovirus to bind to the host epithelial cell surface and prevent infection, and (ii) it reduces immune cell mediated inflammation. Which of these two effects are responsible for the clinically reported reduced viral infections by EPs 7630 has to be investigated in future studies. However, direct evidence for pro-infectious effect of ICOS and ICOSL in virus infection is missing.

The effects of EPs 7630 on the expression of the above named host cells viral docking and defence proteins were more clearly seen when analysed by immunofluorescence then by immunoblotting. Thus, indicating that the compound modified the cell compartmental location of the viral docking and defence proteins within cells rather than inducing *de novo* synthesis.

The observations of modified expression of virus docking proteins in epithelial cells suggest that the application of EPs 7630 over several days may reduce the infection rate of rhinovirus. However, the presented data also indicate that this effect of EPs 7630 may be cell type specific. Further contribution to the beneficial effect of EPs 7630 on host defence against rhinovirus infection is the observation that β-defensin-1 and SOCS1 were up-regulated after 24 hours. These findings suggest that the host cells may be stimulated to execute intracellular killing of rhinovirus more effectively. The data may also explain the observed improvement of survival of the host cells when incubated for 1 day with EPs 7630 before infection with RV16. However, the role of SOCS1 in the defence against rhinovirus infection is unclear and the literature provides examples of pro-infectious effect, as well as for virus protection [46–48].

As described above, β-defensins have been described to reduce viral infections in different cell types. There is no information for the effect of rhinovirus infection on β-defensin-1. In hBEC, RV16 infection slightly reduced the expression of β-defensin-1, but the effect did not achieve significance. In contrast, EPs 7630 up-regulated the expression of β-defensin-1, in the absence and presence of RV16. The increased expression of β-defensin-1 correlated with the decreased infection of hBEC by RV16, but this study could not provide proof of a causal link of both events. Different β-defensins have been described to help with the clearance of viral or bacterial infections without stimulating inflammation, thus, they are interesting targets for the therapy of any infectious disease [48]. In other species, β-defensin-1 has been reported to act as an adjuvant for virus vaccination including influenza, hepatitis virus, herpes virus, and carp virus as summarised by Kalenik et al. [49]. However, the mechanisms of β-defensins by which they protect the host cells against infection have not been well studied.

In conclusion, EPs 7630 reduced viral docking proteins without showing any disease specific effect. These findings support the concept that EPs 7630 application is reducing the susceptibility of RV-infections. The above described molecular biological *in vitro* results may explain the clinically documented anti-viral benefits of EPs 7630 in patients with bronchitis, asthma and COPD. Thus, preventive usage by patients during seasonal virus epidemics may reduce the likelihood of infection as it was reported recently [50].

## Supporting information

S1 File **Figs** (A) Phase-contrast microscopy of primary human bronchial epithelial cells. Epithelial cells show knobble-stone phenotype (day 0). EPs 7630 alone, second row, did not alter the phenotype over 2 days; while RV16 infection significantly reduced cell numbers (3^rd^ row, 1^st^ photograph). When pre-incubated for 1 day with EPs 7630 cell survival was increased after RV16 infection. RV16 infection was documented by immunofluorescence-staining (green). Nuclei stained with DAPI (blue). (B) The effect of time and concentration on EPs 7630-mediated hBEC survival. Bars represent mean ± S.E.M. of six cell lines in each group. Statistics were calculated as Student’s paired t-test. * indicates P<0.05 compared to untreated hBEC.
(PDF)

S2 File **Table A**: Number of surviving human bronchial epithelial cells after RV16 infection for 6 healthy controls (H01-H06), 6 asthma patients (A01-A06), and 6 COPD patients (CD01-CD06). **Table B**: Number of RV16 infected cells for the same patients presented in table A. Mean, S.D. and S.E.M. as well as Student’s t-test were performed by Excel program.
(PDF)

S3 File **Tables A and B**: Optical density values derived from Fig C by image analysis (imageJ). Data is shown for the same patients shown in S2 File. Mean, S.D. and S.E.M. as well as Student’s t-test were performed by Excel program. **Fig C**: Representative Western-blots of ICOS and ICOSL. Protein bands used to calculate optical density values presented in Tables A and B are marked by brackets.
(PDF)

S4 File **Table A**: Optical density values derived from Fig B by image analysis (imageJ). Data is shown for the same patients shown in S2 File. Mean, S.D. and S.E.M. as well as Student’s t-test were performed by Excel program. **Fig B**: Representative Western-blots of C1qR. Protein bands used to calculate optical density values presented in Table A are marked by brackets.
(PDF)

S5 File **Table A**: Optical density values derived from Fig B by image analysis (imageJ). Data is shown for the same patients shown in S2 File. Mean, S.D. and S.E.M. as well as Student’s t-test were performed by Excel program. Fig B: Representative Western-blots of β-defensin1. Protein bands used to calculate optical density values presented in Table A are marked by brackets.
(PDF)

S6 File **Table A**: Optical density values for SOCS1 obtained by cell based ELISA in the same patients shown in S2 File. Mean, S.D. and S.E.M. as well as Student’s t-test were performed by Excel program.
(PDF)

## References

[1] Looi K, Buckley AG, Rigby PJ, Garratt LW, Iosifidis T, Zosky GR, et al Effects of human rhinovirus on epithelial barrier integrity and function in children with asthma. Clin Exp Allergy. 2018 1 19 10.1111/cea.13097 29350877

[2] Wilkinson TMA, Aris E, Bourne S, Clarke SC, Peeters M, Pascal TG, et al A prospective, observational cohort study of the seasonal dynamics of airway pathogens in the aetiology of exacerbations in COPD. Thorax. 2017;72:919–927. 10.1136/thoraxjnl-2016-209023 28432209 PMC5738531

[3] Zheng XY, Xu YJ, Guan WJ, Lin LF. Regional, age and respiratory-secretion-specific prevalence of respiratory viruses associated with asthma exacerbation: a literature review. Arch Virol. 2018 1 11 10.1007/s00705-017-3700-y 29327237 PMC7087223

[4] Ritchie AI, Farne HA, Singanayagam A, Jackson DJ, Mallia P, Johnston SL. Pathogenesis of Viral Infection in Exacerbations of Airway Disease. Ann Am Thorac Soc. 2015 11;12 Suppl 2:S115–S132. 26595727 10.1513/AnnalsATS.201503-151AW

[5] Thibaut HJ, Lacroix C, De Palma AM, Franco D, Decramer M, Neyts J. Toward antiviral therapy/prophylaxis for rhinovirus-induced exacerbations of chronic obstructive pulmonary disease: challenges, opportunities, and strategies. Rev Med Virol. 2016;26:21–33. 10.1002/rmv.1856 26388447 PMC7169185

[6] Bao Y, Gao Y, Koch E, Pan X, Jin Y, Cui X. Evaluation of pharmacodynamics activities of EPs 7630, a special extract from roots of Pelargonium sidoides, in animals models of cough, secretolytic activity and acute bronchitis. Phytomedicine. 2015;22:504–509. 10.1016/j.phymed.2015.03.004 25925973 PMC7172309

[7] Helfer M, Koppensteiner H, Schneider M, Rebensburg S, Forcisi S, Müller C, et al The root extract of the medicinal plant Pelargonium sidoides is a potent HIV-1 attachment inhibitor. PLoS One. 2014;9:e87487 10.1371/journal.pone.0087487 24489923 PMC3906173

[8] Tahan F, Yaman M. Can the Pelargonium sidoides root extract EPs 7630 prevent asthma attacks during viral infections of the upper respiratory tract in children? Phytomedicine. 2013;20:148–50. 10.1016/j.phymed.2012.09.022 23142309

[9] Patiroglu T, Tunc A, Eke Gungor H, Unal E. The efficacy of Pelargonium sidoides in the treatment of upper respiratory tract infections in children with transient hypogammaglobulinemia of infancy. Phytomedicine. 2012;19:958–961. 10.1016/j.phymed.2012.06.004 22809962

[10] Kamin W, Maydannik V, Malek FA, Kieser M. Efficacy and tolerability of EPs 7630 in children and adolescents with acute bronchitis—a randomized, double-blind, placebo-controlled multicenter trial with a herbal drug preparation from Pelargonium sidoides roots. Int J Clin Pharmacol Ther. 2010;48:184–191. 20197012 10.5414/cpp48184

[11] Kamin W, Ilyenko LI, Malek FA, Kieser M. Treatment of acute bronchitis with EPs 7630: randomized, controlled trial in children and adolescents. Pediatr Int. 2012;54:219–226. 10.1111/j.1442-200X.2012.03598.x 22360575

[12] Michaelis M, Doerr HW, Cinatl J Jr. Investigation of the influence of EPs 7630, a herbal drug preparation from Pelargonium sidoides, on replication of a broad panel of respiratory viruses. Phytomedicine. 2011;18:384–386. 10.1016/j.phymed.2010.09.008 21036571 PMC7127141

[13] Theisen LL, Muller CP. EPs 7630 (Umckaloabo®), an extract from Pelargonium sidoides roots, exerts anti-influenza virus activity in vitro and in vivo. Antiviral Res. 2012;94:147–156. 10.1016/j.antiviral.2012.03.006 22475498

[14] Shukla SD, Hansbro PM, Walters EH. Blocking rhinoviral adhesion molecule (ICAM-1): potential to prevent COPD exacerbations. Int J Chron Obstruct Pulmon Dis. 2017;12:1413–1414. 10.2147/COPD.S138612 28546749 PMC5436784

[15] Traub S, Nikonova A, Carruthers A, Dunmore R, Vousden KA, Gogsadze L, et al An anti-human ICAM-1 antibody inhibits rhinovirus-induced exacerbations of lung inflammation. PLoS Pathog. 2013;9:e1003520 10.1371/journal.ppat.1003520 23935498 PMC3731244

[16] Mukhopadhyay S, Malik P, Arora SK, Mukherjee TK. Intercellular adhesion molecule-1 as a drug target in asthma and rhinitis. Respirology. 2014;19:508–513. 10.1111/resp.12285 24689994

[17] Shukla SD, Mahmood MQ, Weston S, Latham R, Muller HK, Sohal SS, et al The main rhinovirus respiratory tract adhesion site (ICAM-1) is upregulated in smokers and patients with chronic airflow limitation (CAL). Respir Res. 2017;18:6 10.1186/s12931-016-0483-8 28056984 PMC5217320

[18] Stanciu LA, Bellettato CM, Laza-Stanca V, Coyle AJ, Papi A, Johnston SL. Expression of programmed death-1 ligand (PD-L) 1, PD-L2, B7-H3, and inducible costimulator ligand on human respiratory tract epithelial cells and regulation by respiratory syncytial virus and type 1 and 2 cytokines. J Infect Dis. 2006;193:404–412. 10.1086/499275 16388488

[19] Nazzal D, Gradolatto A, Truffault F, Bismuth J, Berrih-Aknin S. Human thymus medullary epithelial cells promote regulatory T-cell generation by stimulating interleukin-2 production via ICOS ligand. Cell Death Dis. 2014;5:e1420 10.1038/cddis.2014.377 25210803 PMC4540205

[20] Qian X, Agematsu K, Freeman GJ, Tagawa Y, Sugane K, Hayashi T. The ICOS-ligand B7-H2, expressed on human type II alveolar epithelial cells, plays a role in the pulmonary host defense system. Eur J Immunol. 2006;36:906–918. 10.1002/eji.200535253 16552709

[21] Kajiwara K, Morishima H, Akiyama K, Yanagihara Y. Expression and function of the inducible costimulator ligand B7-H2 in human airway smooth muscle cells. Allergol Int. 2009;58:573–583. 10.2332/allergolint.09-OA-0113 19776675

[22] Kuroki Y, Takahashi M, Nishitani C. Pulmonary collectins in innate immunity of the lung. Cell Microbiol. 2007;9:1871–1879. 10.1111/j.1462-5822.2007.00953.x 17490408

[23] Hu DD, Mai JN, He LY, Li PQ, Chen WX, Yan JJ, et al Glucocorticoids Prevent Enterovirus 71 Capsid Protein VP1 Induced Calreticulin Surface Exposure by Alleviating Neuronal ER Stress. Neurotox Res. 2017;31:204–217. 10.1007/s12640-016-9670-0 27848175

[24] Lee JJ, Seah JB, Chow VT, Poh CL, Tan EL. Comparative proteome analyses of host protein expression in response to Enterovirus 71 and Coxsackievirus A16 infections. J Proteomics. 2011;74:2018–2024. 10.1016/j.jprot.2011.05.022 21621020

[25] Roth M, Pasquali C, Stolz D, Tamm M. Broncho Vaxom (OM-85) modulates rhinovirus docking proteins on human airway epithelial cells via Erk1/2 mitogen activated protein kinase and cAMP. PLoS One. 2017;12:e0188010 10.1371/journal.pone.0188010 29182620 PMC5705076

[26] Witte K, Koch E, Volk HD, Wolk K, Sabat R. The Pelargonium sidoides Extract EPs 7630 Drives the Innate Immune Defense by Activating Selected MAP Kinase Pathways in Human Monocytes. PLoS One. 2015;10:e0138075 10.1371/journal.pone.0138075 26406906 PMC4583277

[27] Wilson SS, Wiens ME, Smith JG. Antiviral mechanisms of human defensins. J Mol Biol. 2013;425:4965–4980. 10.1016/j.jmb.2013.09.038 24095897 PMC3842434

[28] Guaní-Guerra E, Negrete-García MC, Montes-Vizuet R, Asbun-Bojalil J, Terán LM. Human β-defensin-2 induction in nasal mucosa after administration of bacterial lysates. Arch Med Res. 2011;42:189–194. 10.1016/j.arcmed.2011.04.003 21722813

[29] Andresen E, Günther G, Bullwinkel J, Lange C, Heine H. Increased expression of beta-defensin 1 (DEFB1) in chronic obstructive pulmonary disease. PLoS One. 2011;6:e21898 10.1371/journal.pone.0021898 21818276 PMC3139569

[30] Liao JY, Zhang T. [Influence of OM-85 BV on hBD-1 and immunoglobulin in children with asthma and recurrent respiratory tract infection]. Zhongguo Dang Dai Er Ke Za Zhi. 2014;16:508–12. 24857002

[31] Bedke N, Sammut D, Green B, Kehagia V, Dennison P, Jenkins G, et al Transforming growth factor-beta promotes rhinovirus replication in bronchial epithelial cells by suppressing the innate immune response. PLoS One. 2012;7:e44580 10.1371/journal.pone.0044580 22970254 PMC3435262

[32] Gielen V, Sykes A, Zhu J, Chan B, Macintyre J, Regamey N, et al Increased nuclear suppressor of cytokine signaling 1 in asthmatic bronchial epithelium suppresses rhinovirus induction of innate interferons. J Allergy Clin Immunol. 2015;136:177–188.e11. 10.1016/j.jaci.2014.11.039 25630941 PMC4541718

[33] Wickert LE, Karta MR, Audhya A, Gern JE, Bertics PJ. Simvastatin attenuates rhinovirus-induced interferon and CXCL10 secretion from monocytic cells in vitro. J Leukoc Biol. 2014;95:951–959. 10.1189/jlb.0713413 24532643 PMC4021432

[34] Hostettler KE, Roth M, Burgess JK, Gencay MM, Gambazzi F, Black JL, et al Airway epithelium-derived transforming growth factor-beta is a regulator of fibroblast proliferation in both fibrotic and normal subjects. Clin Exp Allergy. 2008;38:1309–1317. 10.1111/j.1365-2222.2008.03017.x 18503568

[35] Oliver BG, Lim S, Wark P, Laza-Stanca V, King N, Black JL, et al Rhinovirus exposure impairs immune responses to bacterial products in human alveolar macrophages. Thorax. 2008;63:519–525. 10.1136/thx.2007.081752 18245149

[36] Kamin W, Funk P, Seifert G, Zimmermann A, Lehmacher W. EPs 7630 is effective and safe in children under 6 years with acute respiratory tract infections: clinical studies revisited. Curr Med Res Opin. 2018;34:475–485. 10.1080/03007995.2017.1402754 29119837

[37] Lambers C, Qi Y, Eleni P, Costa L, Zhong J, Tamm M, Block LH, Roth M. Extracellular matrix composition is modified by β₂-agonists through cAMP in COPD. Biochem Pharmacol. 2014 10 1;91(3):400–8. 10.1016/j.bcp.2014.07.026 25107701

[38] Matthys H, Pliskevich DA, Bondarchuk OM, Malek FA, Tribanek M, Kieser M. Randomised, double-blind, placebo-controlled trial of EPs 7630 in adults with COPD. Respir Med. 2013;107:691–701. 10.1016/j.rmed.2013.02.011 23478193

[39] Humphreys IR, Edwards L, Snelgrove RJ, Rae AJ, Coyle AJ, Hussell T. A critical role for ICOS co-stimulation in immune containment of pulmonary influenza virus infection. Eur J Immunol. 2006;36:2928–2938. 10.1002/eji.200636155 17039567

[40] Wu J, Cui D, Yang X, Lou J, Lin J, Ye X, et al Increased frequency of circulating follicular helper T cells in children with hand, foot, and mouth disease caused by enterovirus 71 infection. J Immunol Res. 2014;2014:651872 10.1155/2014/651872 25013818 PMC4071862

[41] de Haij S, Woltman AM, Trouw LA, Bakker AC, Kamerling SW, van der Kooij SW, et al Renal tubular epithelial cells modulate T-cell responses via ICOS-L and B7-H1. Kidney Int. 2005;68:2091–2102. 10.1111/j.1523-1755.2005.00665.x 16221208

[42] Macaubas C, DeKruyff RH, Umetsu DT. Respiratory tolerance in the protection against asthma. Curr Drug Targets Inflamm Allergy. 2003;2:175–186. 14561171 10.2174/1568010033484304

[43] Liu W, Feng W, Wang F, Li W, Zhou B, Gao C, et al Adenovirus-mediated ICOSIg gene transfer alleviates cardiac remodeling in experimental autoimmune myocarditis. Immunol Cell Biol. 2008;86:659–665. 10.1038/icb.2008.45 19005474

[44] Sato J, Konno N, Murakami M, Uede T, Himi T. Adenovirus-Mediated ICOSIg Gene Therapy in a Presensitized Murine Model of Allergic Rhinitis. Adv Otorhinolaryngol. 2016;77:59–66. 10.1159/000441876 27116360

[45] Blumer T, Coto-Llerena M, Duong FHT, Heim MH. SOCS1 is an inducible negative regulator of interferon λ (IFN-λ)-induced gene expression in vivo. J Biol Chem. 2017;292:17928–17938. 10.1074/jbc.M117.788877 28900038 PMC5663890

[46] Dowran R, Sarvari J, Moattari A, Fattahi MR, Ramezani A, Hosseini SY. Analysis of TLR7, SOCS1 and ISG15 immune genes expression in the peripheral blood of responder and non-responder patients with chronic Hepatitis C. Gastroenterol Hepatol Bed Bench. 2017;10:272–277. 29379591 PMC5758734

[47] Obajemu AA, Rao N, Dilley KA, Vargas JM, Sheikh F, Donnelly RP, et al IFN-λ4 Attenuates Antiviral Responses by Enhancing Negative Regulation of IFN Signaling. J Immunol. 2017;199:3808–3820. 10.4049/jimmunol.1700807 29070670 PMC5698113

[48] Zhang G., Sunkara L.T. Avian antimicrobial host defense peptides: from biology to therapeutic applications. Pharmaceuticals (Basel), 2014;7:220–247 24583933 10.3390/ph7030220 PMC3978490

[49] Kalenik BM, Góra-Sochacka A, Sirko A. Β-defensins—Underestimated peptides in influenza combat. Virus Res. 2018;247:10–14. 10.1016/j.virusres.2018.01.008 29421304

[50] Careddu D, Pettenazzo A. Pelargonium sidoides extract EPs 7630: a review of its clinical efficacy and safety for treating acute respiratory tract infections in children. Int J Gen Med. 2018;11:91–98. 10.2147/IJGM.S154198 29563828 PMC5849386

