# Supplementary figures and images for: Pelargonium sidoides radix extract EPs 7630 reduces rhinovirus infection through modulation of viral binding proteins on human bronchial epithelial cells
Source: PLoS One. 2019 Feb 1;14(2):e0210702. doi: 10.1371/journal.pone.0210702 (PMC6358071; doi:10.1371/journal.pone.0210702)

## S1 A

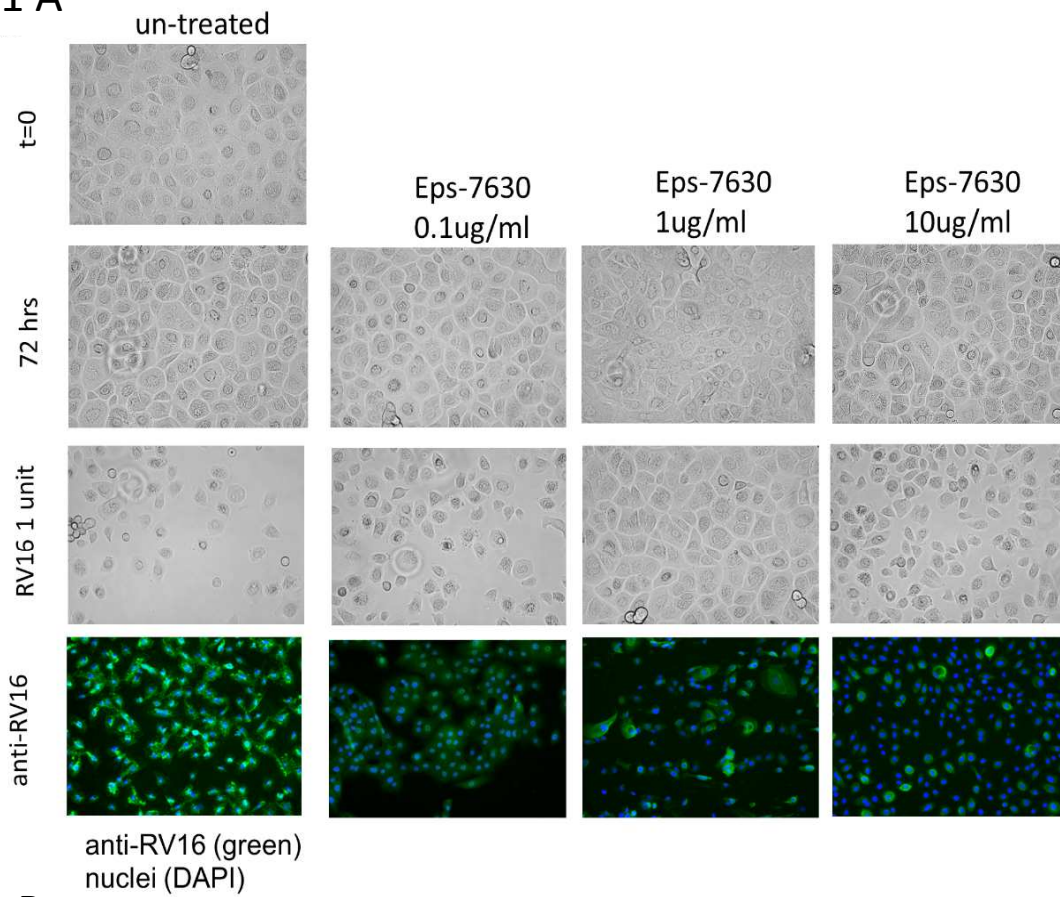

## S1 B

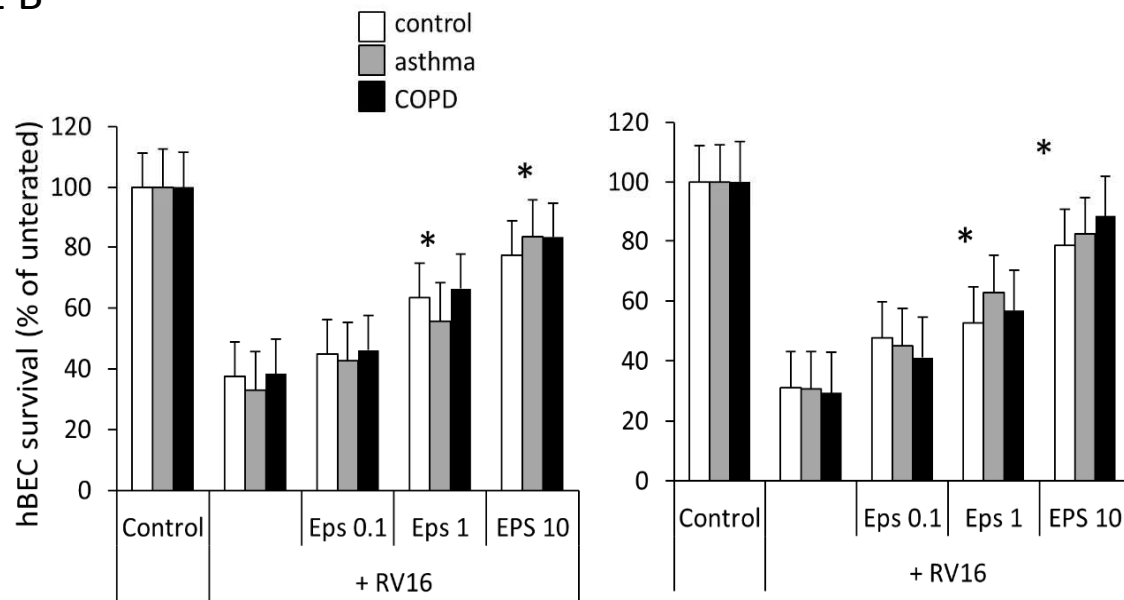

Supplement: S1 File — Figs (A) Phase-contrast microscopy of primary human bronchial epithelial cells. Epithelial cells show knobble-stone phenotype (day 0). EPs 7630 alone, second row, did not alter the phenotype over 2 days; while RV16 infection significantly reduced cell numbers (3rd row, 1st photograph). When pre-incubated for 1 day with EPs 7630 cell survival was increased after RV16 infection. RV16 infection was documented by immunofluorescence-staining (green). Nuclei stained with DAPI (blue). (B) The effect of time and concentration on EPs 7630-mediated hBEC survival. Bars represent mean ± S.E.M. of six cell lines in each group. Statistics were calculated as Student’s paired t-test. * indicates P<0.05 compared to untreated hBEC. (PDF) [file pone.0210702.s001.pdf]

S4 A

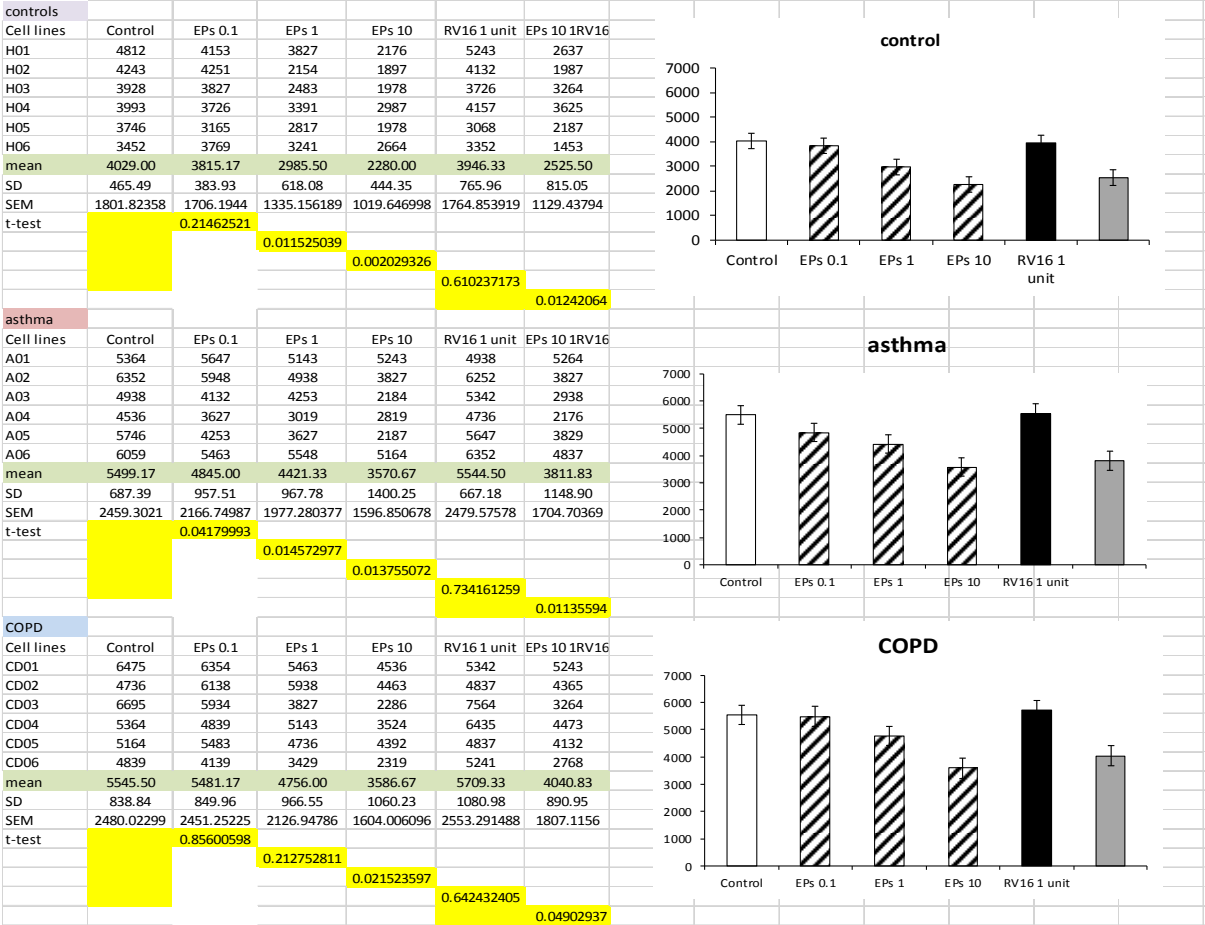

S4 B

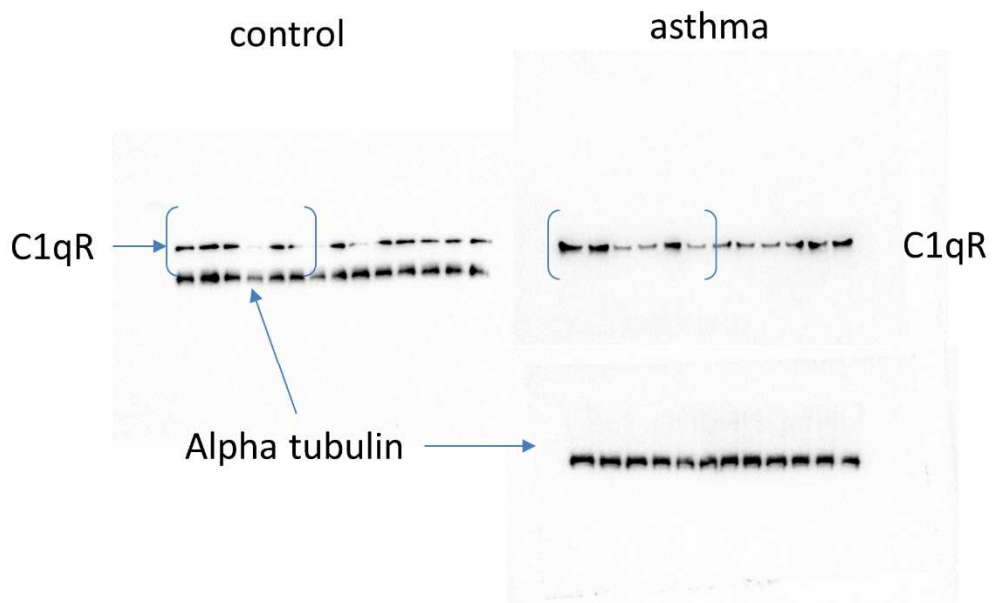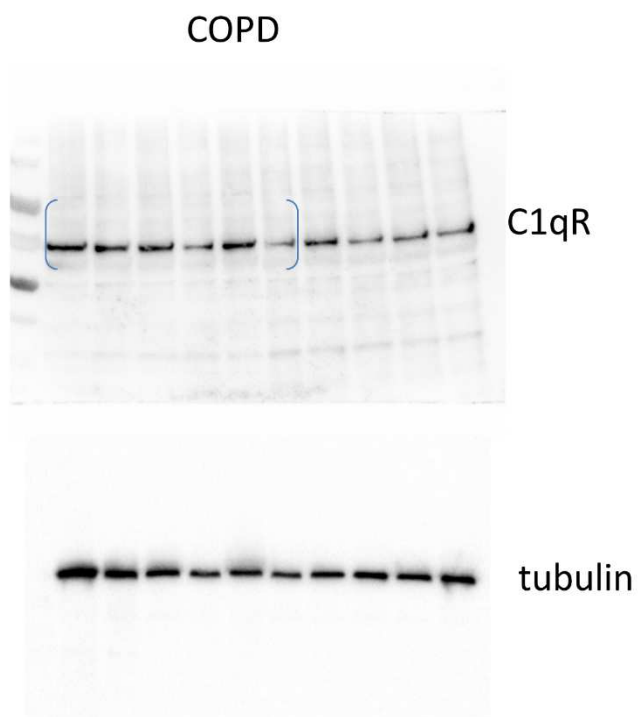

Supplement: S4 File — Table A: Optical density values derived from Fig B by image analysis (imageJ). Data is shown for the same patients shown in S2 File. Mean, S.D. and S.E.M. as well as Student’s t-test were performed by Excel program. Fig B: Representative Western-blots of C1qR. Protein bands used to calculate optical density values presented in Table A are marked by brackets. (PDF) [file pone.0210702.s004.pdf]

# S6 A

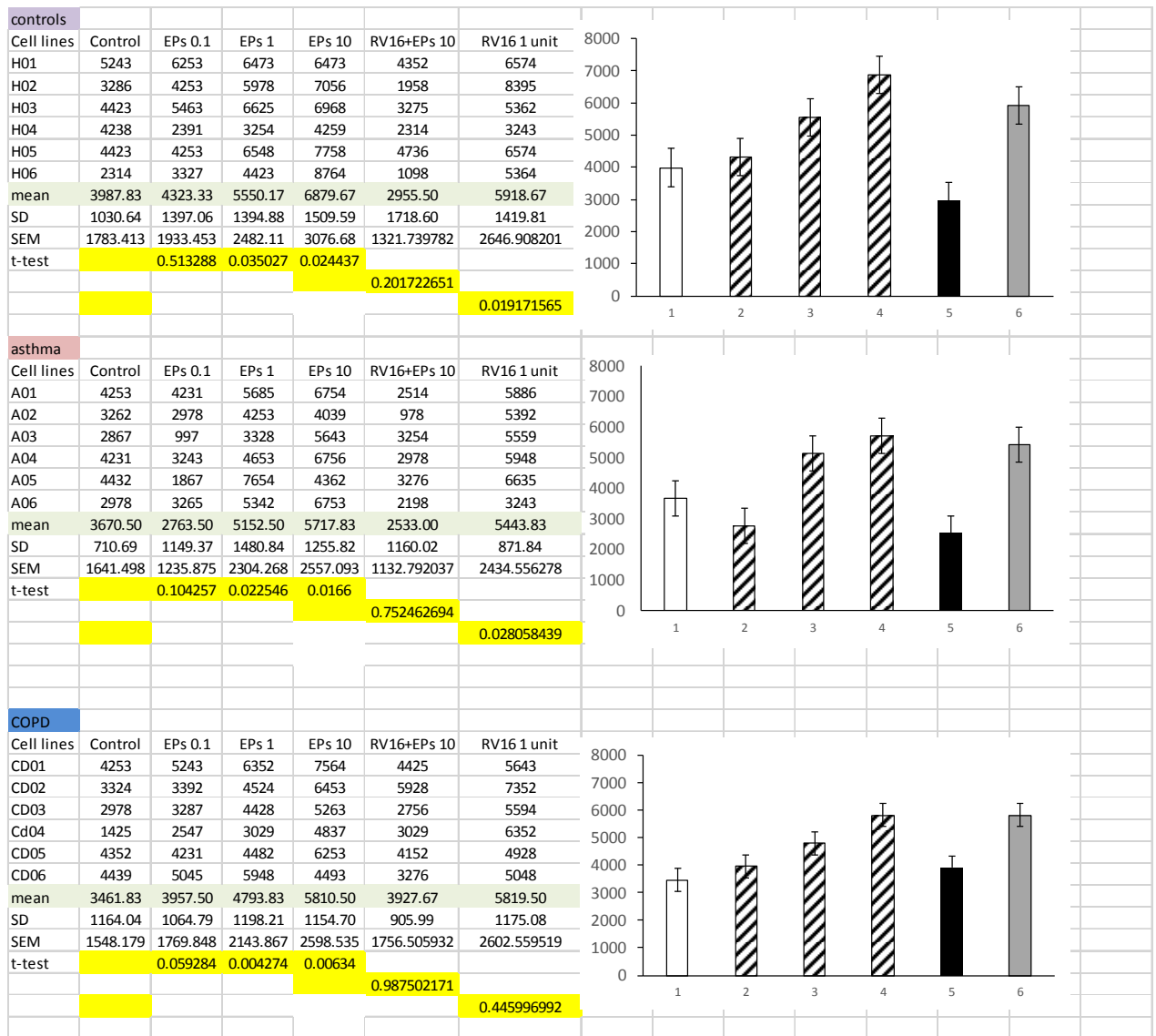

Supplement: S6 File — Table A: Optical density values for SOCS1 obtained by cell based ELISA in the same patients shown in S2 File. Mean, S.D. and S.E.M. as well as Student’s t-test were performed by Excel program. (PDF) [file pone.0210702.s006.pdf]
