# Supplementary material for: Pelargonium sidoides radix extract EPs 7630 reduces rhinovirus infection through modulation of viral binding proteins on human bronchial epithelial cells
Source: PLoS One. 2019 Feb 1;14(2):e0210702. doi: 10.1371/journal.pone.0210702 (PMC6358071; doi:10.1371/journal.pone.0210702)

S2 A

controls	+ RV16				
Cell lines	Control		Eps 0.1	Eps 1	EPS 10
H01	100	53	33	53	75
H02	100	16	0	64	97
H03	100	24	12	75	76
H04	100	53	25	52	86
H05	100	49	53	55	74
H06	100	35	14	76	93
mean	100.00	38.33	22.83	62.50	83.50
SD	0.00	15.87	18.63	10.93	9.97
SEM	44.7213595	17.1431878	10.2113771	27.9508497	37.3423352
t-test		0.00021655			
					0.00461695
				0.06393842	
			0.01790025		

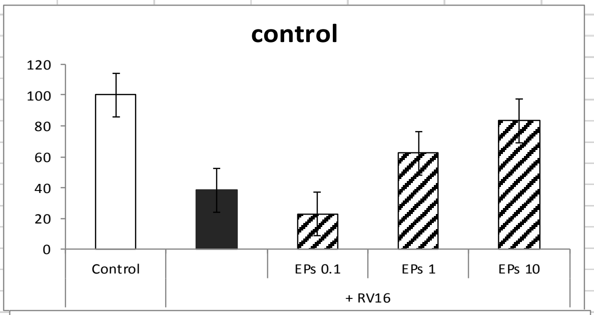

asthma	+ RV16				
Cell lines	Control		Eps 0.1	Eps 1	EPS 10
A01	100	69	21	37	84
A02	100	21	43	93	95
A03	100	34	28	47	52
A04	100	49	61	87	85
A05	100	25	35	53	82
A06	100	34	42	43	68
mean	100.00	38.67	38.33	60.00	77.67
SD	0.00	17.72	13.91	23.89	15.27
SEM	44.7213595	17.292259	17.1431878	26.8328157	34.7335893
t-test		0.0003747			
					0.00857782
				0.19041286	
			0.97523456		

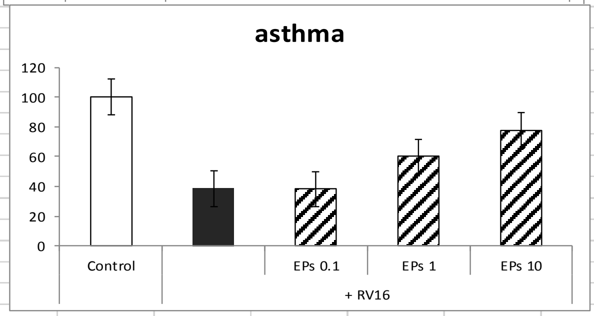

COPD	+ RV16				
Cell lines	Control		Eps 0.1	Eps 1	EPS 10
CD01	100	37	27	56	85
CD02	100	25	43	57	94
CD03	100	58	19	43	75
CD04	100	36	28	43	98
CD05	100	41	56	64	72
CD06	100	25	83	42	57
mean	100.00	37.00	42.67	50.83	80.17
SD	0.00	12.21	23.77	9.37	15.25
SEM	44.7213595	16.546903	19.0811134	22.7333578	35.8516232
t-test		5.5198E-05			
					0.00323675
				0.09223635	
			0.69044594		

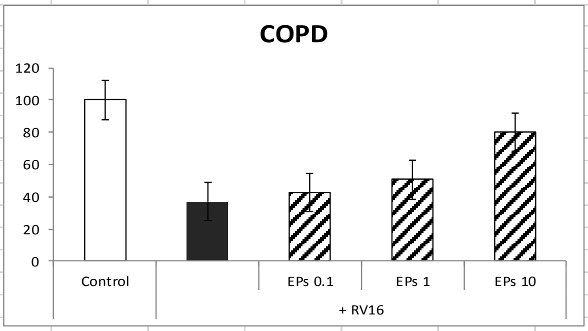

S2 B

	+ RV16			
Control		EPs 0.1	EPs 1	EPs 10
3624	15342	14837	11253	10958
4253	16453	16003	16453	9476
3142	14258	15542	12746	11726
5264	15329	14253	14352	13246
6473	15649	16278	15534	11823
3241	13241	14253	12134	10058
4332.83	15045.33	15194.33	13745.33	11214.50
1308.92	1130.48	877.39	2034.98	1355.33
1937.70197	6728.47762	6795.11244	6147.09994	5015.27687
	3.0766E-06			
				0.00305741
			0.08519665	
		0.71775837		

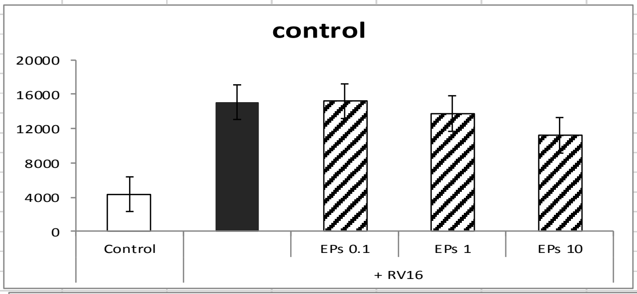

	+ RV16			
Control		EPs 0.1	EPs 1	EPs 10
3827	11869	11521	10237	7584
4425	15243	15648	14239	9568
5938	12134	13286	13247	11425
1425	14960	13243	13287	10853
6312	14352	14342	15344	11028
4938	16453	15342	14638	9685
4477.50	14168.50	13897.00	13498.67	10023.83
1758.08	1814.42	1536.13	1788.74	1409.58
2002.39887	6336.34583	6214.92734	6036.78725	4482.79455
	0.00032798			
				0.00458976
			0.28270086	
		0.54868644		

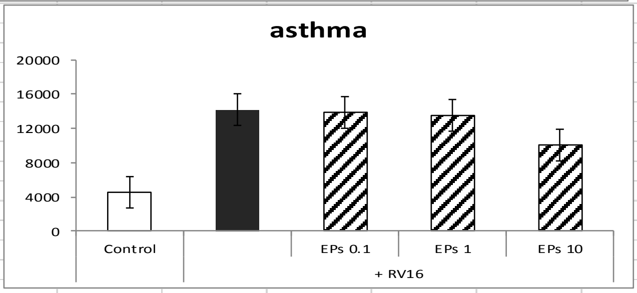

	+ RV16			
Control		EPs 0.1	EPs 1	EPs 10
4132	11859	12748	7563	9485
1758	15243	15342	11057	11049
3726	12638	11947	14233	8475
1197	16043	14382	15243	9186
6253	14296	13456	9968	11723
3274	15263	12827	14253	10275
3390.00	14223.67	13450.33	12052.83	10032.17
1808.11	1645.63	1231.93	3008.91	1215.89
1516.05409	6361.01711	6015.17193	5390.19093	4486.52133
	0.00031658			
				0.00158078
			0.08444945	
		0.17248595		

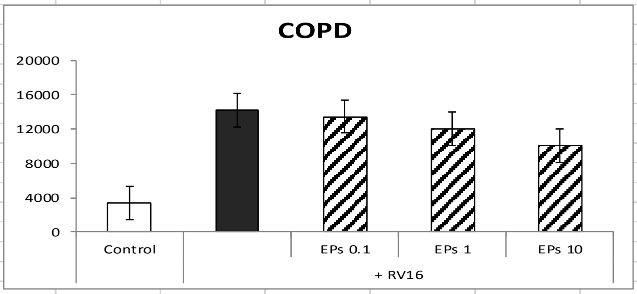

Supplement: S2 File — Table A: Number of surviving human bronchial epithelial cells after RV16 infection for 6 healthy controls (H01-H06), 6 asthma patients (A01-A06), and 6 COPD patients (CD01-CD06). Table B: Number of RV16 infected cells for the same patients presented in table A. Mean, S.D. and S.E.M. as well as Student’s t-test were performed by Excel program. (PDF) [file pone.0210702.s002.pdf]
