# Supplementary material for: Pelargonium sidoides radix extract EPs 7630 reduces rhinovirus infection through modulation of viral binding proteins on human bronchial epithelial cells
Source: PLoS One. 2019 Feb 1;14(2):e0210702. doi: 10.1371/journal.pone.0210702 (PMC6358071; doi:10.1371/journal.pone.0210702)

S3 A

controls						
Cell lines	Control	EPS 0.1	EPs 1	EPs 10	RV16 1 unit	EPs 10 + RV16
H01	2754	2785	1987	1128	4648	2165
H02	4463	4132	3124	1097	4352	2253
H03	4058	3928	2978	1987	4287	2314
H04	3827	4231	2736	1752	4938	1978
H05	3928	4402	3892	3928	5463	3142
H06	4635	4355	3243	1645	4152	2197
mean	3944.17	3972.17	2993.33	1922.83	4640.00	2341.50
SD	661.80	605.83	627.08	1043.55	492.32	408.30
SEM	1763.88496	1776.40694	1338.65936	859.917209	2075.07108	1047.15063
t-test		0.84948768				
			0.00075195			
				0.00282328		
					0.07411238	

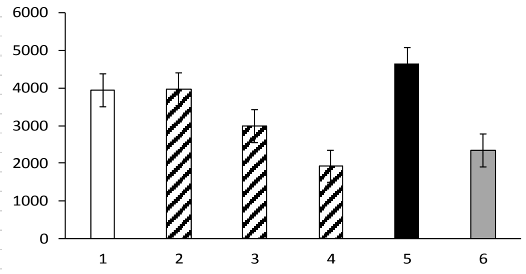

asthma						
Cell lines	Control	EPS 0.1	EPs 1	EPs 10	RV16 1 unit	EPs 10 + RV16
A01	3545	3827	3142	1165	4837	3241
A02	4038	3948	2879	1897	4153	3187
A03	4123	3621	2341	3214	4438	2534
A04	3625	2879	2546	1542	3264	2978
A05	7465	6475	3425	5463	8675	4473
A06	4736	4839	3187	1876	4352	2987
mean	4588.67	4264.83	2920.00	2526.17	4953.17	3233.33
SD	1471.94	1252.33	412.89	1596.12	1896.62	656.34
SEM	2052.11412	1907.29145	1305.8637	1129.73608	2215.12347	1445.99063
t-test		0.17457877				
			0.01811091			
				0.0077381		
					0.1220985	

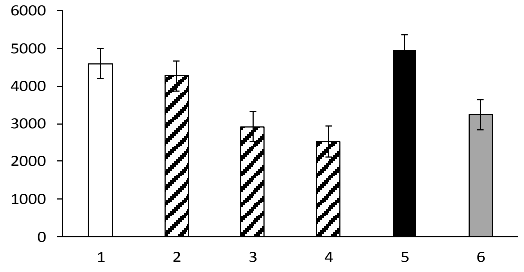

COPD						
Cell lines	Control	EPS 0.1	EPs 1	EPs 10	RV16 1 unit	EPs 10 + RV16
CD01	3524	3428	1867	1287	3241	2263
CD02	3276	3392	2134	1198	4253	2314
CD03	4938	4351	3264	3386	5164	3129
CD04	4155	4006	3197	2187	4473	2768
CD05	4653	4251	3246	1657	4352	2439
CD06	3928	3376	2413	1425	3827	1756
mean	4079.00	3800.67	2686.83	1856.67	4218.33	2444.83
SD	640.12	454.77	625.91	828.56	645.99	468.45
SEM	1824.18426	1699.70981	1201.5884	830.326576	1886.49602	1093.36271
t-test		0.05878175				
			0.00014647			
				0.00033201		
					0.05299386	

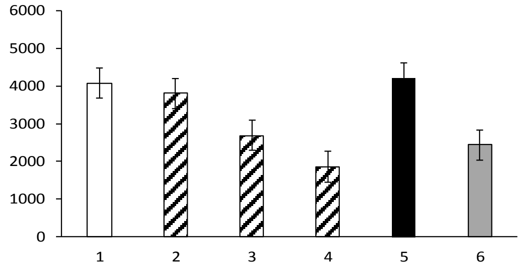

S3 B

controls						
Cell lines	Control	EPS 0.1	EPs 1	EPs 10	RV16 1 unit	EPs 10 + RV16
H01	5364	5243	4837	1103	5465	2154
H02	4938	4837	3265	1978	5324	2657
H03	4235	4132	3186	1243	4635	2736
H04	5547	5647	5186	4938	5189	4472
H05	3948	3827	2635	1876	3726	2164
H06	4465	4498	2312	1282	4837	2435
mean	4749.50	4697.33	3570.17	2070.00	4862.67	2769.67
SD	638.31	683.51	1175.93	1175.93	636.65	868.29
SEM	2124.04097	2100.71133	1596.62707	925.732143	2174.65064	1238.63259
t-test		0.2351643				
			0.00979291			
				0.00979291		
					0.33171122	

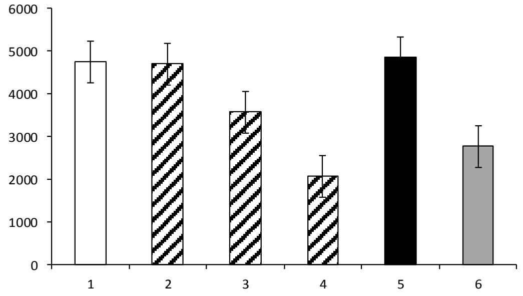

asthma						
Cell lines	Control	EPS 0.1	EPs 1	EPs 10	RV16 1 unit	EPs 10 + RV16
A01	4463	4236	3101	2345	4536	2176
A02	5243	5486	4132	1978	5148	2637
A03	4938	4627	3386	1275	5124	2176
A04	4473	4187	2978	1536	4461	1978
A05	4176	3876	2154	1553	3987	2187
A06	5349	5432	5198	5231	5143	5049
mean	4773.67	4640.67	3491.50	2319.67	4733.17	2700.50
SD	473.79	677.53	1052.48	1052.48	482.19	1170.93
SEM	2134.84863	2075.36923	1561.44627	1037.38647	2116.73648	1207.70031
t-test		0.22695399				
			0.00232154			
				0.00232154		
					0.53221399	

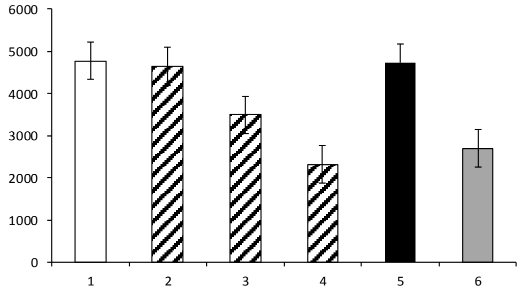

COPD						
Cell lines	Control	EPS 0.1	EPs 1	EPs 10	RV16 1 unit	EPs 10 + RV16
CD01	5364	5243	3726	2187	5546	2078
CD02	4637	4198	3187	1978	4425	2341
CD03	5594	5328	5039	1324	5867	1352
CD04	4736	4635	3625	3127	4352	3274
CD05	3827	4137	2197	1276	4198	1645
CD06	3142	2876	3197	3019	3352	3286
mean	4550.00	4402.83	3495.17	2151.83	4623.33	2329.33
SD	927.35	901.06	929.80	929.80	928.24	811.71
SEM	2034.82186	1969.00693	1563.08605	962.329122	2067.61752	1041.70954
t-test		0.21665179				
			0.04197428			
				0.04197428		
					0.13254909	

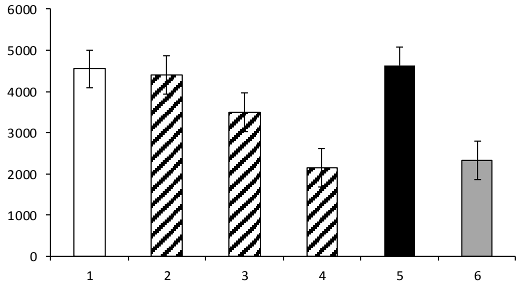

S3 C

Western-blots figure 3, displayed bands are indicated by brakets. Other bands were run for different treatment which are not trelevatnt to this manuscript.

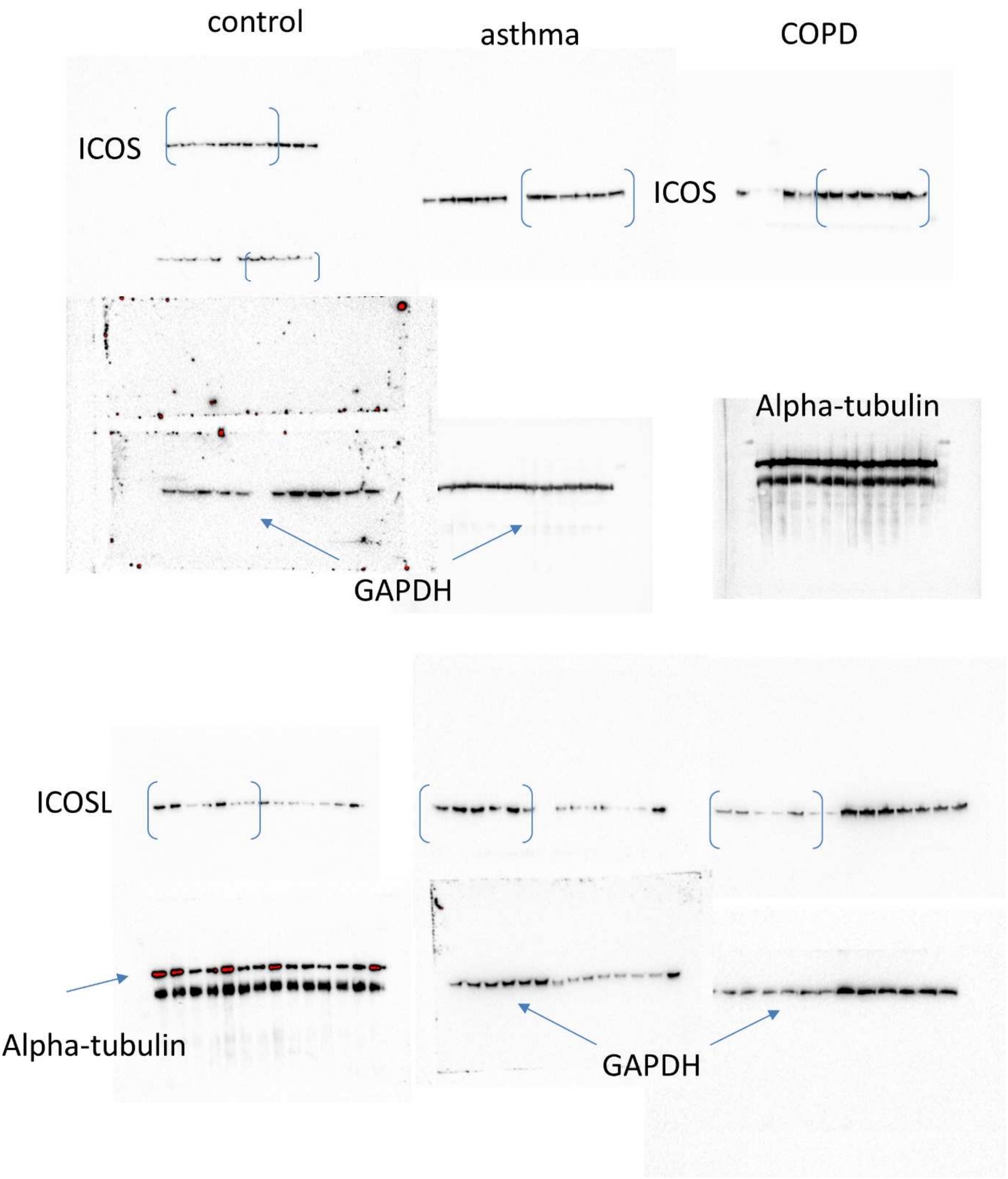

Supplement: S3 File — Tables A and B: Optical density values derived from Fig C by image analysis (imageJ). Data is shown for the same patients shown in S2 File. Mean, S.D. and S.E.M. as well as Student’s t-test were performed by Excel program. Fig C: Representative Western-blots of ICOS and ICOSL. Protein bands used to calculate optical density values presented in Tables A and B are marked by brackets. (PDF) [file pone.0210702.s003.pdf]
