# Supplementary material for: Pelargonium sidoides radix extract EPs 7630 reduces rhinovirus infection through modulation of viral binding proteins on human bronchial epithelial cells
Source: PLoS One. 2019 Feb 1;14(2):e0210702. doi: 10.1371/journal.pone.0210702 (PMC6358071; doi:10.1371/journal.pone.0210702)
Supplement: S5 File — Table A: Optical density values derived from Fig B by image analysis (imageJ). Data is shown for the same patients shown in S2 File. Mean, S.D. and S.E.M. as well as Student’s t-test were performed by Excel program. Fig B: Representative Western-blots of β-defensin1. Protein bands used to calculate optical density values presented in Table A are marked by brackets. (PDF) [file pone.0210702.s005.pdf]

S5 A

controls						
Cell lines	Control	EPs 0.1	EPs 1	EPs 10	RV16+EPs 10	RV16 1 unit
H01	14524	14253	16123	16754	13726	16253
H02	16453	15234	13256	17827	15386	17038
H03	14328	14938	14993	15546	14232	16254
H04	13298	12978	13276	16756	12879	15345
H05	16453	15627	16574	18765	14253	17564
H06	14425	13287	14432	15643	12875	16895
mean	14913.50	14386.17	14775.67	16881.83	13891.83	16558.17
SD	1271.46	1074.42	1398.07	1248.28	776.25	955.61
SEM	6669.51996	6433.68932	6607.87902	7549.78538	6212.616733	7405.03725
t-test		0.11780506	0.84312823	0.00278154		
					0.492840477	
						0.0223531
					0.002001213	

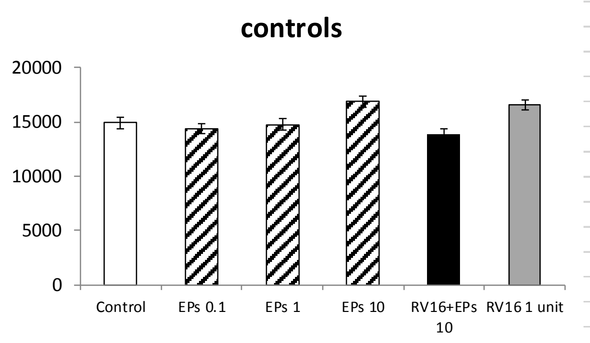

asthma						
Cell lines	Control	EPs 0.1	EPs 1	EPs 10	RV16 1 unit	RV16+EPs 10
A01	10293	11625	12437	15647	12867	18276
A02	11176	10976	11526	13298	11627	16542
A03	12625	13254	14237	16453	12918	16743
A04	13276	13285	16754	18765	10786	15532
A05	14436	13928	14453	14493	12431	14284
A06	12987	13243	14938	16129	13327	15948
mean	12465.50	12718.50	14057.50	15797.50	12326.00	16220.83
SD	1497.98	1146.97	1858.47	1861.05	1334.15	950.41
SEM	5574.74107	5687.88611	6286.70512	7064.85677	5512.354778	7254.1772
t-test		0.38796837	0.02746439	0.0107165		
					0.673938556	
						0.8608037
					0.021370315	

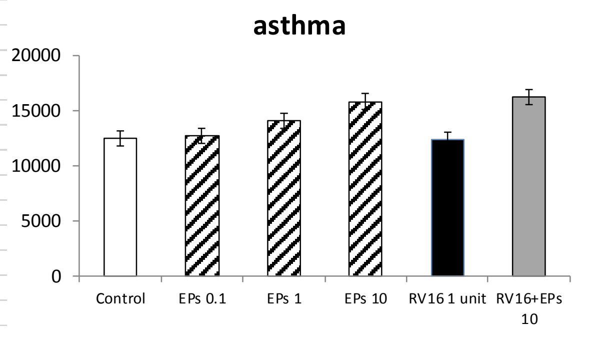

COPD						
Cell lines	Control	EPs 0.1	EPs 1	EPs 10	RV16+EPs 10	RV16 1 unit
CD01	12847	12524	16253	17625	13243	15643
CD02	11093	12978	16483	16948	12413	17265
CD03	13287	12775	14263	15342	14236	15495
Cd04	12423	11293	13928	13328	13297	16354
CD05	12294	13253	15243	17265	11524	14938
CD06	11964	12764	14423	14993	12756	15039
mean	12318.00	12597.83	15098.83	15916.83	12911.50	15789.00
SD	755.82	684.22	1076.57	1654.37	882.30	917.68
SEM	5508.77707	5633.92234	6752.40354	7118.22426	5774.198338	7061.05546
t-test		0.56771994	0.00730463	0.0058178		
					0.877152653	
						0.10334685
					0.001996698	

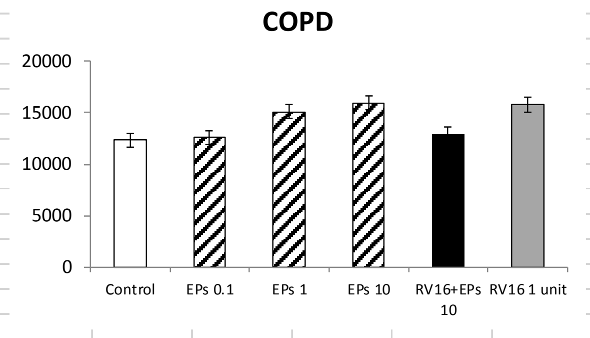

S5 B

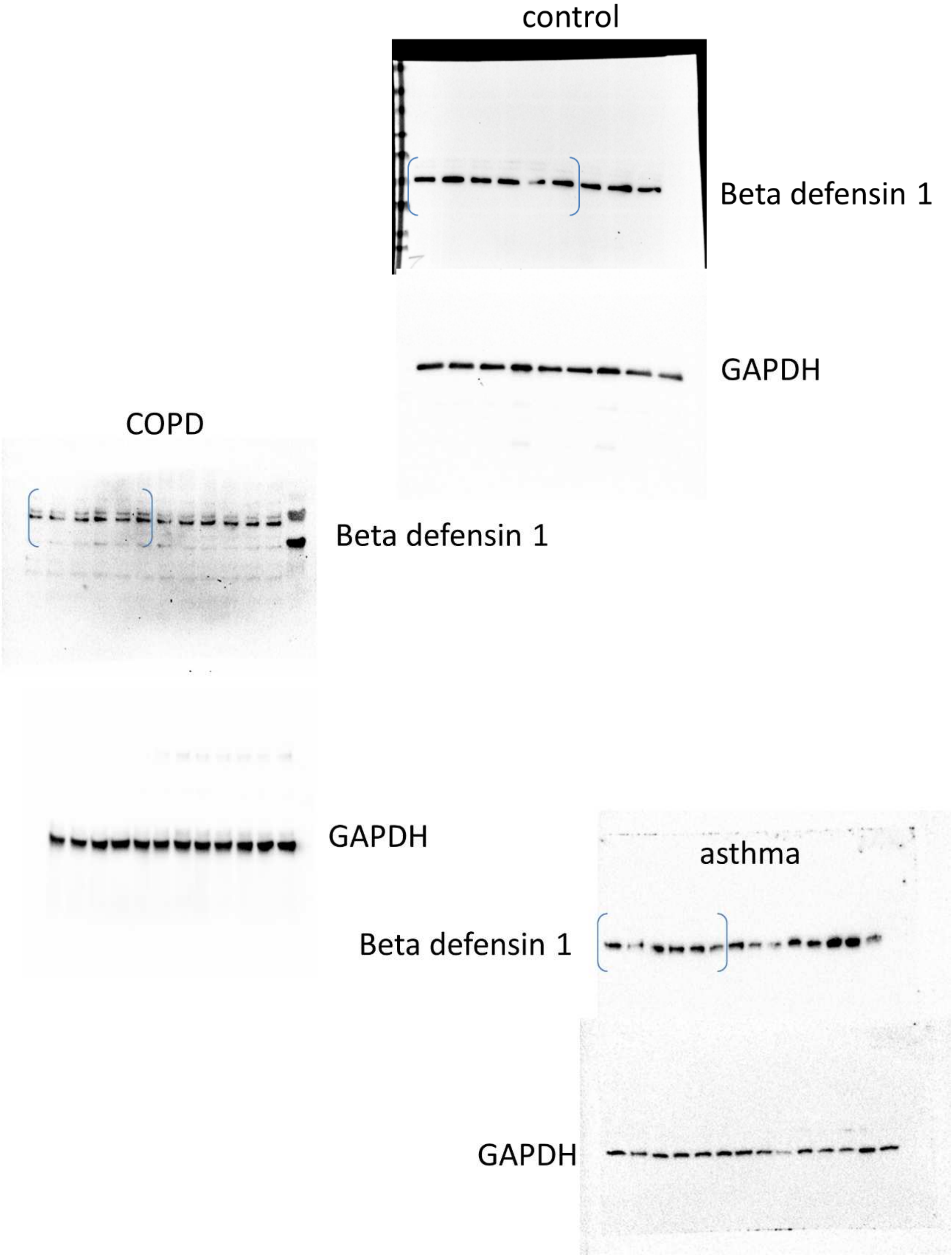
control
Beta defensin 1
GAPDH
COPD
Beta defensin 1
GAPDH
asthma
Beta defensin 1
GAPDH
